# Engineered IL-7 synergizes with IL-12 immunotherapy to prevent T cell exhaustion and promote memory without exacerbating toxicity

**DOI:** 10.1126/sciadv.adh9879

**Published:** 2023-11-29

**Authors:** Seounghun Kang, Aslan Mansurov, Trevin Kurtanich, Hye Rin Chun, Anna J. Slezak, Lisa R. Volpatti, Kevin Chang, Thomas Wang, Aaron T. Alpar, Kirsten C. Refvik, O. Isabella Hansen, Gustavo J. Borjas, Ha-Na Shim, Kevin T. Hultgren, Suzana Gomes, Ani Solanki, Jun Ishihara, Melody A. Swartz, Jeffrey A. Hubbell

**Affiliations:** ^1^ Pritzker School of Molecular Engineering, University of Chicago, Chicago, IL, USA.; ^2^ Committee on Immunology, University of Chicago, Chicago, IL, USA.; ^3^ Animal Resource Center, University of Chicago, Chicago, IL, USA.; ^4^ Ben May Department for Cancer Research, University of Chicago, Chicago, IL, USA.; ^5^ Committee on Cancer Biology, University of Chicago, Chicago, IL, USA.; ^6^ Department of Bioengineering, Imperial College London, London, UK.

## Abstract

Cancer immunotherapy is moving toward combination regimens with agents of complementary mechanisms of action to achieve more frequent and robust efficacy. However, compared with single-agent therapies, combination immunotherapies are associated with increased overall toxicity because the very same mechanisms also work in concert to enhance systemic inflammation and promote off-tumor toxicity. Therefore, rational design of combination regimens that achieve improved antitumor control without exacerbated toxicity is a main objective in combination immunotherapy. Here, we show that the combination of engineered, tumor matrix-binding interleukin-7 (IL-7) and IL-12 achieves remarkable anticancer effects by activating complementary pathways without inducing any additive immunotoxicity. Mechanistically, engineered IL-12 provided effector properties to T cells, while IL-7 prevented their exhaustion and boosted memory formation as assessed by tumor rechallenge experiments. The dual combination also rendered checkpoint inhibitor (CPI)–resistant genetically engineered melanoma model responsive to CPI. Thus, our approach provides a framework of evaluation of rationally designed combinations in immuno-oncology and yields a promising therapy.

## Teaser

Dual therapy of engineered IL-7 and IL-12 variants led to synergistic antitumor efficacy without inducing additive immunotoxicity.

## INTRODUCTION

While immuno-oncology has become one of the dominant pillars of modern cancer care, single-agent immunotherapies still demonstrate low response rates against certain types of cancers (*1*–*3*). To increase response rates and overcome resistance to single-agent immunotherapy, combinatorial treatment with complimentary agents has emerged as a promising approach (*4*–*6*). For example, combining checkpoint inhibitor (CPI) antibodies blocking the programmed cell death protein 1 (PD-1) (e.g., with nivolumab) and cytotoxic T-lymphocyte-associated protein 4 (CTLA-4) (e.g., ipilimumab) axes increases the overall survival of patients with melanoma when compared to blockade with either agent alone (*7*–*9*). However, a major limitation of combination immunotherapies is that the rate of immune-related adverse events (irAEs) also increases when compared to the monotherapy regimens (*2*, *10*, *11*). In the same trial examining the combination of nivolumab and ipilimumab, 55 to 60% of patients experienced grade 3 or 4 adverse events, while monotherapy led to only 10 to 20% of patients experiencing grade 3 or 4 adverse events. A similar observation was made in another trial examining interleukin-12 (IL-12) in combination with IL-2 (*12*). Thus, rational selection of agents that act on distinct axes and enhance clinical outcomes without exacerbating overall toxicity is highly desired.

IL-7 is a common gamma chain (γ_c_) cytokine that plays a crucial role in T cell development, expansion, and memory (*13*–*16*). IL-7 signals through IL-7 receptor α (IL-7Rα) and γ_c_, the former being predominantly expressed on mature T cells and dictating the cell-type specificity of this cytokine. Because of the immunomodulatory properties of IL-7, it has been tested in a clinical trial in patients with advanced cancers as monotherapy (*17*). IL-7 was well-tolerated up to a dose of 60 μg/kg and induced robust expansion of peripheral CD8^+^ T cells, but not regulatory T cells (T_regs_) or natural killer (NK) cells (*18*–*20*). Despite demonstrating obvious biological activity at well-tolerated doses, IL-7 did not show any clinical benefit in a monotherapy setting. A possible explanation for the lack of tumor regression is that IL-7 by itself does not induce potent effector functions in CD8^+^ T cells (*21*) and would likely require a combination approach involving an immune-stimulatory factor to fully unlock its therapeutic potential.

Here, we designed a combination therapy approach consisting of IL-7 and an immune-stimulatory cytokine, IL-12, which augments effector properties of T cells. We used an engineered form for both cytokines to improve their biodistribution, leveraging our reported tumor stroma–binding technology, which uses a collagen-binding domain (CBD) derived from the A3 domain of von Willebrand factor as a tumor retention domain (*22*–*25*). We show that the combination of IL-7–CBD with CBD–IL-12 addresses a key challenge in combination immunotherapy: increased therapeutic efficacy without compromised safety. We demonstrate this result by conducting extensive toxicology studies and paralleling those with the antitumor efficacy studies of the IL-7–CBD + CBD–IL-12 combination therapy. We also show that IL-7–CBD and CBD–IL-12 activate nonredundant immunological pathways, with IL-7–CBD providing the memory and survival signals and CBD–IL-12 supporting the effector programs of the tumor-resident T cells. IL-7–CBD prevented the conversion of activated T cells into exhausted ones, resulting in functional, long-term immune memory as evidenced by the lack of tumor recurrence. In addition, the IL-7–CBD and CBD–IL-12 combination therapy overcame resistance to PD-1 blockade in an orthotopic triple-negative breast cancer model and in a genetically engineered melanoma model. By contrast, use of engineered IL-15 to enhance memory with CBD–IL-12 was associated with substantially elevated toxicity. Together, our studies reveal a basis for the selection criteria of rationally designed combination immunotherapies.

## RESULTS

### IL-7–CBD synergizes with CBD–IL-12 without increasing systemic toxicity

**Fig. 1.**
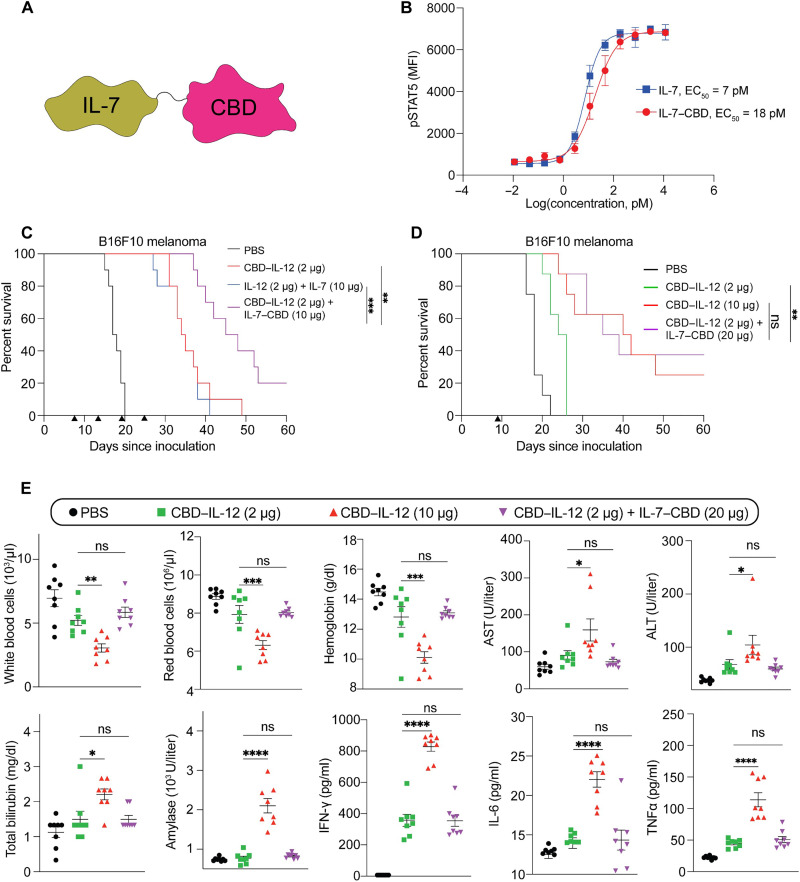
In vitro characterization of IL-7–CBD and intravenously administrated CBD–IL-12 and IL-7–CBD combination therapy exhibit synergistic antitumor effect and reduce toxicity compared to high dose of CBD–IL-12 (10 μg). (**A**) Molecular schematic illustration indicating the fusion of CBD to the C terminus of recombinant IL-7. (**B**) Dose response of phosphorylated STAT5 with mouse IL-7 and IL-7–CBD fusion protein in CD3^+^ T cells. MFI, mean fluorescence intensity; EC_50_, median effective concentration. (**C**) B16F10-bearing mice were treated with either PBS, 33.3 pmol of IL-12 + 666 pmol of IL-7, 33.3 pmol of CBD–IL-12, or 33.3 pmol of CBD–IL-12 + 666 pmol of IL-7–CBD intravenously on days 7, 13, 19, and 25. Survival curves (C) are shown. (**D**) B16F10-bearing mice were treated with either PBS, 33.3 pmol of CBD–IL-12, 166.5 pmol of CBD–IL-12, or 33.3 pmol of CBD–IL-12 + 1332 pmol of IL-7–CBD intravenously on day 9. Survival curves (D) are shown. (**E**) C57BL/6 mice were injected with PBS, 33.3 pmol of CBD–IL-12, 166.5 pmol of CBD–IL-12, or 33.3 pmol of CBD–IL-12 + 1332 pmol of IL-7–CBD intravenously, and blood was collected on day 2 for cytokine expression level analysis (IFN-γ, IL-6, and TNF-α), on day 3 for blood chemistry analysis (AST, ALT, amylase, and total bilirubin), and on day 4 for circulating blood cell count (white blood cells, red blood cells, and hemoglobin). Overall survival rates were compiled for two independent experiments, and statistical analyses were performed using log-rank (Mantel-Cox) tests. Blood toxicity analysis was compiled for multiple comparisons, and statistical analyses were performed using one-way analysis of variance (ANOVA) tests. *P < 0.05, **P < 0.01, ***P < 0.001, and ****P < 0.0001; ns, not significant.

Motivated by the improved therapeutic properties of intravenously or intratumorally administered immunotherapy agents when fused to the CBD derived from the A3 domain of von Willebrand factor (CBD) (*22*), which leads to active retention in the exposed tumor stroma, we fused the CBD to the C terminus (IL-7–CBD), N terminus (CBD–IL-7), or C and N termini (CBD–IL-7–CBD) of murine IL-7 and chose to proceed with IL-7–CBD, which had a higher yield (5.8 mg/liter) than other candidates (CBD–IL-7, 4.8 mg/liter; and CBD–IL-7–CBD, 3 mg/liter) (Fig. 1A and fig. S1A). C-terminal fusion of CBD did not abrogate the activity of IL-7 as assessed by signal transducer and activator of transcription 5 (STAT5) phosphorylation on mouse T cells (Fig. 1B). We then performed a collagen-coated enzyme-linked immunosorbent assay to confirm that IL-7–CBD has adequate binding to collagen I, as we have observed with other cytokine fusions to CBD (fig. S1B) (*23*). Last, we performed an in vivo tumor growth inhibition study using the MC38 colorectal adenocarcinoma model, which is known to respond to IL-7 treatment (*26*), to compare the antitumor activity of IL-7 versus IL-7–CBD (fig. S2). In line with our previous observations, intravenous administration of an equimolar dose of IL-7–CBD significantly extended the survival of MC38-bearing mice when compared to the unmodified IL-7. Collectively, these data indicate that the fusion of CBD to IL-7 retains both IL-7 receptor-stimulating activity and collagen-binding properties, as well as improves the monotherapy efficacy in vivo.

After demonstrating the superior antitumor efficacy of IL-7–CBD over unmodified IL-7 in the MC38 model, which is known to be CPI-responsive (*27*, *28*), we sought to combine it with CBD–IL-12 (*23*) and assess the efficacy of the combination therapy in the CPI-unresponsive B16F10 melanoma model (Fig. 1C and fig. S3). In comparison with the unmodified IL-7 + IL-12, CBD-fused cytokine combination therapy significantly increased the survival when administered intravenously. To understand the therapeutic benefit of the addition of IL-7–CBD to CBD–IL-12, we designed an experiment in which mice received either a 10-μg CBD–IL-12 monotherapy (166.5 pmol, or 10 μg on an IL-12 basis) or a 2-μg CBD–IL-12 (33.3 pmol, or 2 μg on an IL-2 basis) + IL-7–CBD combination therapy (Fig. 1D and fig. S4). A single intravenous administration of a 10-μg CBD–IL-12 monotherapy and a 2-μg CBD–IL-12 + IL-7–CBD combination therapy yielded equally significant extension in the survival of B16F10 melanoma–bearing mice when compared to phosphate-buffered saline (PBS). This result indicates that we are able to reduce the dose of CBD–IL-12 fivefold by supplementing the CBD–IL-12 regimen with IL-7–CBD at no detriment to antitumor efficacy. We also observed that escalating the dose of IL-7–CBD proportionally increases the antitumor efficacy when combined with CBD–IL-12, indicating that synergistic therapeutic efficacy is dependent on the dose of IL-7–CBD (fig. S5).

A key hurdle in the majority of combination therapy trials is that, despite the overall increased survival, such combination approaches often lead to higher rates of irAEs. To investigate whether addition of IL-7–CBD to CBD–IL-12 worsens the tolerability of the therapy, we performed extensive toxicology studies in mice receiving either a 10-μg CBD–IL-12 monotherapy, a 2-μg CBD–IL-12 monotherapy, or a combination of 2 μg of CBD–IL-12 and 20 μg of IL-7–CBD systemically (Fig. 1E). We collected the blood of treated mice at various time points to quantify the levels of circulating leukocytes, erythrocytes, organ damage markers (alanine aminotransferase, aspartate aminotransferase, amylase, and total bilirubin) and proinflammatory cytokines [interferon-γ (IFN-γ), IL-6, and tumor necrosis factor–α (TNF-α)]. Our data demonstrate that IL-7–CBD + CBD–IL-12–treated animals experienced these irAEs similar to 2-μg (based on IL-12 molar equivalents) CBD–IL-12 monotherapy–treated animals, which was significantly lower than in the 10-μg CBD–IL-12–treated cohort. This lack of stimulation of systemic inflammation upon IL-7 combination with IL-12 was likely an IL-7–specific phenomenon because the combination of IL-15, another actively studied γ_c_ cytokine, with IL-12 led to significantly higher systemic inflammation and toxicity (fig. S6). To further evaluate toxicity, we performed a single-dose or multiple-dose toxicity test in tumor-bearing mice (figs. S7 and S8), where the dose and injection interval were based on the single-dose or multiple-dose therapeutic evaluation (Fig. 1, C and D). The CBD–IL-12 monotherapy and the IL-7–CBD + CBD–IL-12 combination therapy induced liver toxicity in both single-dose and multiple-dose injections, but the combination therapy showed toxicity similar to the 2-μg CBD–IL-12 monotherapy, and IL-7–CBD in the combination therapy did not lead to the additional irAEs. To demonstrate that the CBD-fused cytokine combination was superior and safer than the unmodified cytokine combination, we designed pharmacokinetic and toxicity studies (figs. S9 to S11). In line with our published results with IL-12 forms in monotherapy (*23*, *29*), the IL-7–CBD + CBD–IL-12 combination therapy exhibited short systemic circulation and promoted tumor accumulation via tumor stroma targeting of the CBD (fig. S12) and induced fewer systemic side effects, including less hepatic CD8^+^ T cell infiltration, versus unmodified IL-7 + IL-12 therapy (fig. S13). Together, these results indicate that IL-7–CBD and CBD–IL-12 display a significant synergy in promoting antitumor immunity without increasing irAEs in healthy organs.

### Addition of IL-7–CBD to CBD–IL-12 therapy reduces the frequency of terminally exhausted CD8^+^ T cells

**Fig. 2.**
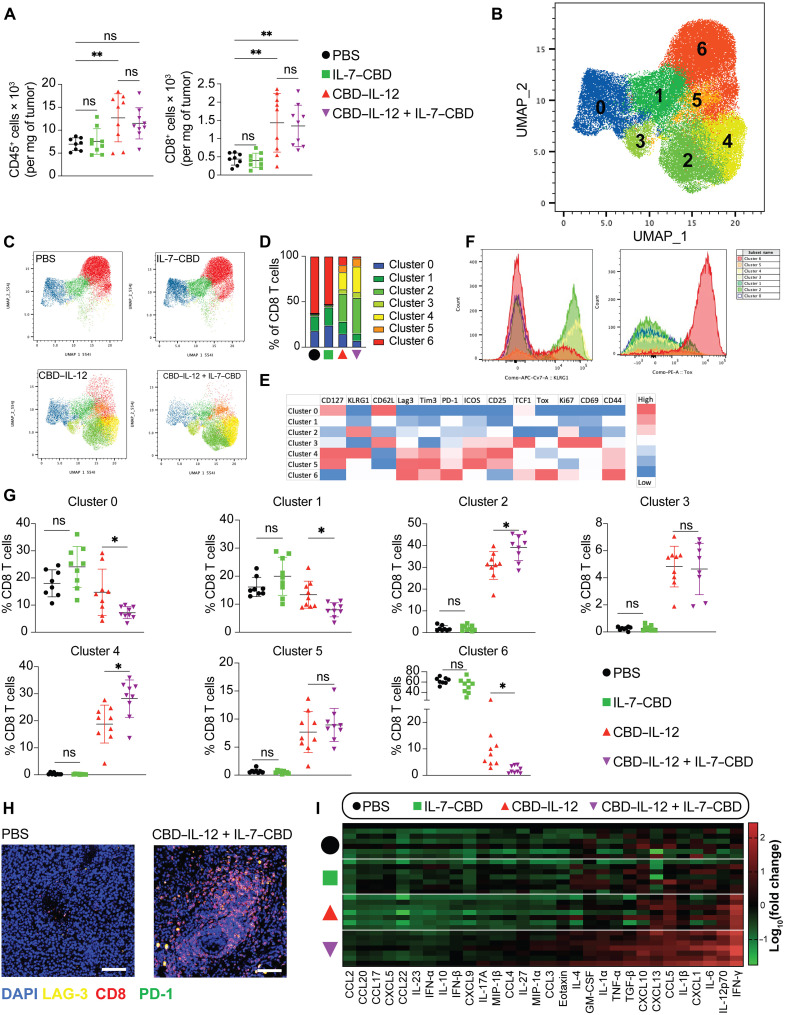
IL-7–CBD synergizes with CBD–IL-12 to antagonize CD8^+^ T cell exhaustion and promote CD8^+^ effector T cells but does not alter CD8^+^ T cell infiltration, and CBD–IL-12 and IL-7–CBD combination therapy induces intratumoral inflammation. B16F10-bearing mice were treated with either PBS, 33.3 pmol of CBD–IL-12, 333 pmol of IL-7–CBD, or 33.3 pmol of CBD–IL-12 + 333 pmol of IL-7–CBD intratumorally on day 7, and tumors were harvested day 13. Cells were digested into a single-cell suspension, stained, and run by flow cytometry. (**A**) Overall counts of CD45^+^ and CD8^+^ T Cells. (**B**) Uniform Manifold Approximation and Projection (UMAP) of concatenated CD8^+^ T cells with FlowSom clustering displayed as an overlay. (**C**) UMAP displaying individual groups as labeled with clusters overlaid. (**D**) Stacked bar graph displaying the percentage of each group per cluster. (**E**) Heatmap of FlowSom clusters. (**F**) Expression of KLRG1 and Tox for each FlowSom cluster. (**G**) Percent population of each cluster for each sample. (**H**) The tumors were harvested after day 6 of the injection and performed with a paraffin embedding process for CD8, PD-1, and LAG-3 fluorescence staining. (**I**) The tumors were homogenized for protein extraction, and cytokine or chemokine expression levels were quantified using LEGENDplex and normalized by total protein content. Statistical analyses were performed using one-way ANOVA tests. *P < 0.05 and **P < 0.01. GM-CSF, granulocyte-macrophage colony-stimulating factor.

To understand the mechanism of action and the therapeutic benefit of the addition of IL-7–CBD to CBD–IL-12, we performed spectral flow cytometry to provide an in-depth look at the tumor-infiltrating lymphocytes. CBD–IL-12 induced an increase in total CD45^+^ immune cell infiltration (Fig. 2A), but the addition of IL-7–CBD did not further increase this. Furthermore, CBD–IL-12 increases conventional (Foxp3^−^) CD4^+^ T cell counts, although not significantly, and decreases T_reg_ infiltration, but IL-7–CBD did not further alter either of these populations in the combination therapy (fig. S14). Neither IL-7–CBD nor CBD–IL-12 monotherapies altered NK or NK T cell infiltration, but combination therapy decreased both NK and NKT cell infiltration compared to the CBD–IL-12 monotherapy (fig. S14).

In terms of CD8^+^ T cell infiltration, addition of IL-7–CBD did not alter CD8^+^ T cell infiltration either when added as a monotherapy, compared to untreated mice, or as a combination therapy when compared to the CBD–IL-12 monotherapy (Fig. 2A). However, because CD8^+^ T cells are considered so important for immunotherapy responses and can be so heterogenous, we next assessed whether the specific phenotype of the CD8^+^ T cell infiltrates was altered. We performed unsupervised clustering using FlowSom, an algorithm specifically developed to analyze flow cytometry data, on CD8^+^ T cells. We observed seven distinct clusters as determined by the algorithm, and they segregated distinctly when displayed on the Uniform Manifold Approximation and Projection (UMAP) of the same CD8^+^ T cells (Fig. 2B). The dimensionality-reduced data demonstrate that, while IL-7–CBD monotherapy does not seem to alter the CD8^+^ T cell phenotype significantly compared to PBS treatment, combining it with CBD–IL-12 alters the T cell landscape due to distinct distribution of the cells in each cluster (Fig. 2C). Specifically, clusters 2, 4, and 6 were significantly influenced by the IL-7–CBD combination when compared against the CBD–IL-12 monotherapy (Fig. 2D). Cluster 6 expressed high levels of canonical exhaustion markers such as T cell immunoglobulin and mucin domain 3 (Tim3) and PD-1 (*30*, *31*), and it also uniquely expressed high levels of Tox, a marker known to be associated with terminal exhaustion (Fig. 2E) (*32*). This indicates that supplementing CBD–IL-12 therapy with IL-7–CBD significantly reduces the frequency of exhausted CD8^+^ T cells. Contrastingly, clusters 2 and 4 both express high levels of killer cell lectin like receptor G1 (KLRG1), a marker of effector function (Fig. 2, E and F) (*33*). In addition, cluster 4 has higher expression of IL-7Rα (CD127) as well as activation markers such as CD44, CD25, and inducible costimulator (ICOS) and higher expression of PD-1, lymphocyte-activation gene 3 (Lag3), and Tim3 compared to cluster 2. Both clusters 2 and 4 are distinctly enriched upon CBD–IL-12 treatment, but, upon the addition of IL-7–CBD, these clusters are enriched even further and constitute about 70% of total CD8^+^ T cells (Fig. 2G) Thus, the IL-7–CBD + CBD–IL-12 combination therapy alters the CD8^+^ T cell landscape as evidenced by reduced abundance of the terminally exhausted cluster 6 and increased abundance of the “effector-like” clusters 2 and 4.

We also visualized the presence of CD8^+^ T cells throughout the tumor stroma (Fig. 2H) after IL-7–CBD + CBD–IL-12 therapy, further corroborating our abovementioned flow cytometric findings. We then sought to characterize the landscape of intratumoral inflammatory cytokines and chemokines upon treatment with the combination of IL-7–CBD + CBD–IL-12 (Fig. 2I). Consistent with flow cytometry data, IL-7–CBD monotherapy did not result in substantial inflammation when compared to saline treatment. Although CBD–IL-12 monotherapy–treated mice had significant elevation of certain markers, combination with IL-7–CBD increased the breadth and the magnitude of the cytokine secretion within the tumor. Notably, compared with the CBD–IL-12 monotherapy, the normalized amount of intratumoral IFN-γ was higher in the dual-therapy–treated animals (fig. S15). Contrasting this result with the level of circulating IFN-γ (Fig. 1E) indicates that addition of IL-7–CBD boosts only tumor-specific inflammation but not systemic inflammation. To verify whether the combination therapy required immune cell migration from tumor-draining lymph nodes or whether immune cells in the circulation were sufficient to elicit an antitumoral immune response, we evaluated the antitumoral effect of combination therapy in mice injected with FTY720, a sphingosine-1-phosphate receptor modulator that sequesters lymphocytes in lymph nodes (fig. S16). Intratumoral treatment with IL-7–CBD + CBD–IL-12 led to a similar extension in survival in mice receiving FTY720 or vehicle control, pointing out that tumor-resident T cells are sufficient to drive the antitumor response.

### Locally administered IL-7–CBD + CBD–IL-12 generates potent systemic immunity and effective long-term immune response

**Fig. 3.**
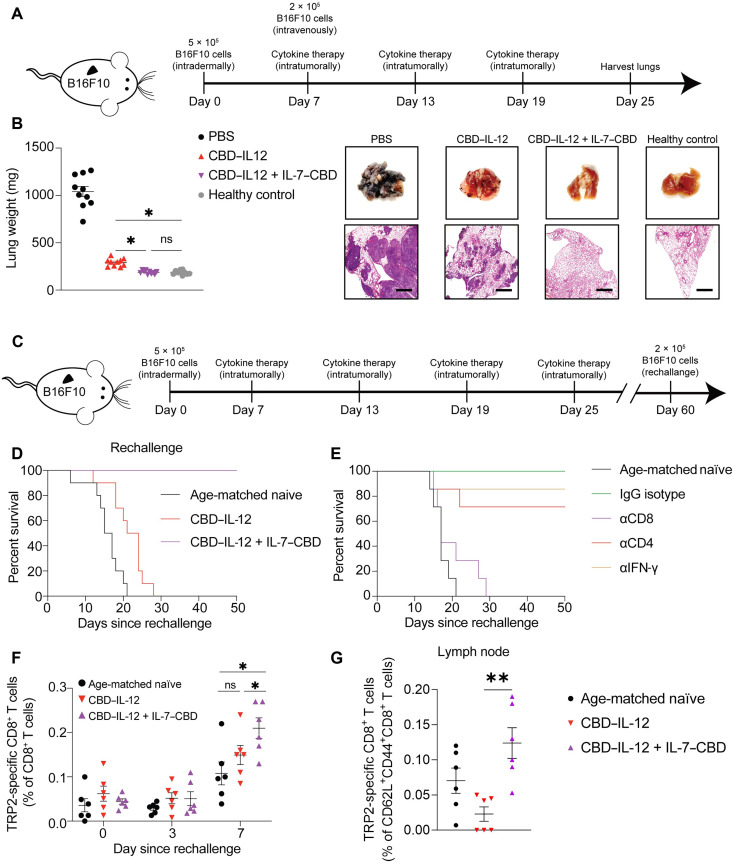
Intratumorally administered CBD–IL-12 and IL-7–CBD combination therapy inhibits lung metastasis of B16F10 and B16F10 rechallenge via an induced systemic tumor-specific immune response. (**A**) B16F10-bearing mice were administrated with 2 × 10^5^ B16F10 intravenously on day 7 and treated with either PBS, 33.3 pmol of CBD–IL-12 or 33.3 pmol of CBD–IL-12 + 666 pmol of IL-7–CBD intratumorally on days 7, 13, and 19. (**B**) The lungs were harvested for measurement of metastasis nodules on day 25. (**C** and **D**) B16F10-free mice cured with 166.5 pmol of CBD–IL-12 [intratumorally (i.t.)] or with 33.3 pmol of CBD–IL-12 + 666 pmol of IL-7–CBD (i.t.) were rechallenged with 2 × 10^5^ B16F10 melanoma intradermally. (**E**) B16F10-free mice due to 33.3-pmol CBD–IL-12 + 666-pmol IL-7–CBD combination therapeutic schedule were rechallenged with 2 × 10^5^ B16F10 and injected with either immunoglobulin G (IgG) isotype, mouse CD8 antibody, mouse CD4 antibody, or mouse IFN-γ antibody on days −1, 2, 5, and 8. (**F**) The tumor-free mice due to 33.3-pmol CBD–IL-12 + 666-pmol IL-7–CBD combination were rechallenged with 2 × 10^5^ B16F10, and the blood was collected on the scheduled time point on days 0, 3, and 7 for quantification of the melanoma antigen (TRP2)–specific CD8^+^ T cells in the circulating lymphocyte. (**G**) The primary tumor-draining lymph nodes were harvested on day 11 for quantification of the melanoma antigen (TRP2)–specific CD62L^+^CD44^+^CD8^+^ T cells. Overall survival rates were compiled for two independent experiments, and statistical analyses were performed using log-rank (Mantel-Cox) tests. Melanoma lung metastasis nodules and TRP2-specific CD8^+^ T cells analysis was compiled for multiple comparisons, and statistical analyses were performed using one-way ANOVA tests or *t* test. *P < 0.05 and **P < 0.01.

Intratumoral administration of immunotherapeutics has emerged as an alternative to systemic administration, especially for intratumorally accessible cancers, so as to limit the systemic exposure and reduce side effects (*34*–*36*). Given the fusion of the CBD to IL-7 and IL-12, local administration may provide further advantages in certain indications in a clinical setting due to the retention of the cytokines in the tumor matrix. We first compared the therapeutic efficacy of the intratumoral injection of CBD-fused cytokine combination therapy to the unmodified IL-7 + IL-12 in the B16F10 melanoma model (fig. S17). CBD-fused cytokine combination therapy yielded significantly extended survival of the melanoma-bearing mice compared to the unmodified combination, with an 80% complete response (CR) rate. The extension in survival can be attributed to the prolonged intratumoral residence of the cytokines (fig. S18). We then tested local administration of monotherapy of CBD–IL-12 or IL-7–CBD, compared to the combination, which was superior, at around 70% CR in this instance (fig. S19). It is crucial for locally administered immunotherapies not only to work against the primary tumors but also to produce systemic antitumor effects. To investigate this, we designed an experiment in which the primary B16F10 tumor was treated at the same time as an intravenous challenge of B16F10 cells, which are reported to colonize the lungs (Fig. 3A) (*37*). Intratumoral treatment with CBD–IL-12 alone did not completely abrogate the formation of such metastases, but therapy with IL-7–CBD + CBD–IL-12 combination completely prevented colonization of the lungs as evidenced by the lung weight and hematoxylin and eosin (H&E) staining, which were not different from the healthy lungs (Fig. 3B).

Because IL-7 plays a major role in generation of immunological memory, we next sought to dissect the contribution of the IL-7–CBD portion in the IL-7–CBD + CBD–IL-12 combination therapy and compare it with the immune memory established by the CBD–IL-12 monotherapy. To match the CR rate of the CBD–IL-12 monotherapy to the IL-7–CBD + CBD–IL-12 combination therapy, we used a fivefold higher dose (10 μg on an IL-12 basis) of CBD–IL-12 than the dose used in the combination regimen. Both the 10-μg CBD–IL-12 monotherapy and the IL-7–CBD + CBD–IL-12 combination therapy led to about 80% CR rate against the primary B16F10 tumor (fig. S20). We then rechallenged these mice on the contralateral side of the back 60 days after the primary tumor inoculation (Fig. 3C). All mice whose primary tumors were cured by the 10-μg CBD–IL-12 monotherapy succumbed to this rechallenge with B16F10 melanoma cells (Fig. 3D). In notable contrast, all of the mice that were initially cured by the combination regimen rejected the rechallenge, indicating that long-term immune memory was established against poorly immunogenic B16F10 melanoma. Next, we performed a depletion study using αCD8, αCD4, and αIFN-γ to identify the immune players that mediate rejection of the rechallenge in combination-treated animals (Fig. 3E). The results indicate that CD8^+^ T cells, but not CD4^+^ T cells or IFN-γ, have the most profound effect on the prevention of regrowth of the B16F10 tumor.

We hypothesized that, upon a rechallenge with B16F10 cells, there is an expansion of tumor-specific memory CD8^+^ T cells, which mediates the rejection of the delayed contralateral challenge. To study this, we evaluated the changes in the population of CD8^+^ T cells specific to tyrosinase-related protein 2 (TRP2), a well-known B16F10 melanoma antigen, in the blood and primary tumor-draining lymph nodes in the cured mice. Mice that were cured by the combination therapy showed significantly greater expansion of TRP2-specific CD8^+^ T cells in the blood compared to CBD–IL-12 monotherapy–cured mice 7 days after reinoculation (Fig. 3F). In addition, when looking at the tumor-draining lymph node, there were more TRP2-specific CD8^+^ T cells that were positive for the canonical central memory markers CD62L and CD44 in mice cured by the combination therapy versus the 10-μg CBD–IL-12 monotherapy (Fig. 3G). These data demonstrate that intratumorally administered IL-7–CBD combined with CBD–IL-12 could boost systemic antitumoral immune response and inhibit pulmonary metastasis and could promote tumor-specific anamnestic response, especially tumor-specific CD8^+^ T cells, and prevent tumor regrowth.

### Combining IL-7–CBD + CBD–IL-12 regimen with αPD-1 elicits long-term tumor control of immunosuppressive breast tumors and genetically engineered melanoma

**Fig. 4.**
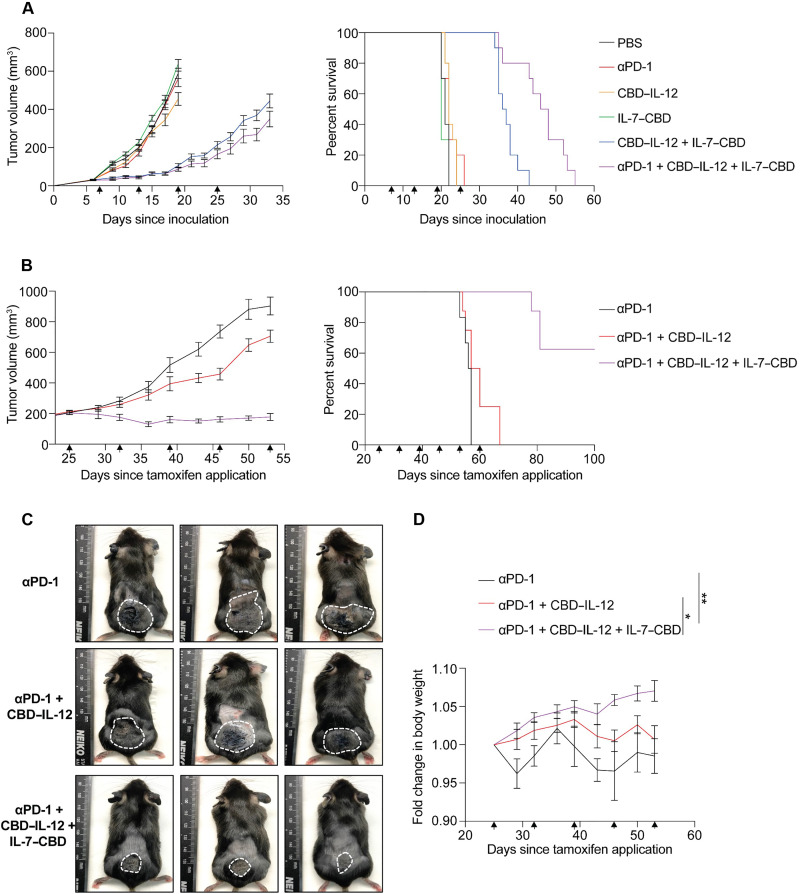
IL-7–CBD + CBD–IL-12 combination therapy synergizes with CPI and effectively suppresses tumor growth even for the tumors with low objective responsive rate to αPD-1 immunotherapy such as 4T1 triple-negative breast cancer and inducible Braf^V600E^/Pten^−/−^/βCAT^STA^ melanoma models. (**A**) Balb/C mice were inoculated with 5 × 10^5^ 4T1 breast cancer cells on mammary pad and treated with either PBS (i.t.), 100 μg of CPI [intraperitoneal (i.p.)], 33.3 pmol of CBD–IL-12 (i.t.), 666 pmol of IL-7–CBD (i.t.), 33.3 pmol of CBD–IL-12 + 666 pmol of IL-7–CBD (i.t.), or 100 μg of CPI + 33.3 pmol of CBD–IL-12 + 666 pmol of IL-7–CBD (i.t.) on days 7, 13, 19, and 25. The tumor growth curve and overall survival rate are shown (A). (**B** to **D**) To demonstrate the superiority of the intravenous IL-7–CBD + CBD–IL-12 combination therapy, Braf^V600E^/Pten^−/−^/βCAT^STA^ mice were applied 50 μg of 4-Hydroxy(OH)-tamoxifen on the back skin and treated with either 100 μg of CPI (i.p.), 100 μg of CPI (i.p.) + 33.3 pmol of CBD–IL-12 [intravenous (i.v.)], or 100 μg of CPI (i.p.) + 33.3 pmol of CBD–IL-12 + 666 pmol of IL-7–CBD (i.v.). The tumor size growth curve overall survival rate (B), the photograph of melanoma induced mice (C), and body weight exchange graph of therapeutic approach mice (D) are shown. Mice body weight analysis on day 53 was compiled for multiple comparisons, and statistical analyses were performed using one-way ANOVA tests. *P < 0.05 and **P < 0.01.

CPI therapy targeting PD-1 is a representative immuno-oncology approach and has led to favorable results in the treatment of certain carcinomas. However, the number of reports that show insufficient therapeutic index in poorly inflamed (or cold) tumors is continuously increasing (*38*–*40*). To investigate whether IL-7–CBD + CBD–IL-12 dual therapy can synergize with αPD-1 immunotherapy, we designed an experiment in which mice received intravenous cytokines of either of the single agents, or the dual therapy, or the triple combination with αPD-1 in the orthotopic 4T1 highly immunosuppressive breast cancer model (Fig. 4A and fig. S21). Neither of the tested monotherapies nor αPD-1 monotherapy resulted in any therapeutic benefit when compared to the saline treatment. The dual engineered cytokine therapy significantly extended the survival of 4T1-bearing animals, corroborating the synergistic effect between IL-7–CBD and CBD–IL-12. Upon further addition of αPD-1 antibody to the dual therapy regimen, mice experienced even greater extension in the overall survival, highlighting that all three agents act on nonredundant immune pathways. Then, we further investigated the CPI potentiating effect of the dual therapy in the genetically engineered Braf^V600E^/Pten^−/−^/βCAT^STA^ melanoma, an immune-desert and CPI-resistant model (Fig. 4, B to D, and fig. S22) (*41*). Systemic administration of the triple therapy led to notable, long-term tumor control, without any loss in body weight during the course of the treatment. Together, our data indicate that combining IL-7–CBD + CBD–IL-12 dual therapy with αPD-1 can overcome CPI resistance and deliver remarkable therapeutic outcomes in very challenging and clinically relevant cancer models.

## DISCUSSION

Combinatorial immunotherapy is a promising approach that can overcome the limitations of monotherapy by enhancing overall survival rate. However, a major hurdle of the combination therapies is the increased cumulative toxicity. Hence, current immunotherapy combinations benefit only those patients who can tolerate the additional side effects. To achieve a net gain in the therapeutic index, it is desired to identify immunotherapy agents with complementary mechanisms of action that do not elicit an additive toxicity profile.

In this study, a dual therapy involving tumor stroma–binding IL-7 and IL-12 variants is used to guide the design of next-generation immunotherapy combinations, which lead to synergistic antitumor efficacy without compromised tolerability. Systemic treatment with IL-7–CBD and CBD–IL-12 significantly improved the survival of poorly immunogenic B16F10 melanoma–bearing mice when compared to either agent in monotherapy. IL-7–CBD did not induce additional irAEs in the combination therapy. Local treatment with IL-7–CBD and CBD–IL-12 induced an antitumor immunity not only at the injection site but also systemically, leading to the eradication of pulmonary B16F10 metastases. During combination therapy, IL-7–CBD played a key role in the development of immunological memory and prevented B16F10 melanoma regrowth upon rechallenge. Combination of IL-7–CBD and CBD–IL-12 significantly extended the survival in the 4T1 triple-negative breast cancer model, which was completely resistant to either agent alone. Addition of αPD-1 to the dual therapy regimen further increased the response rates, demonstrating the importance of activating multiple immunological pathways to boost antitumor immunity.

IL-7–CBD provided prosurvival signals mediated by STAT5 activity to tumor-resident T cells, while CBD–IL-12 provided immune-activating signals mediated by STAT4 activation. Potent immune stimulators such as IL-12 may lead to the induction of exhausted T cells (*42*, *43*), which are no longer able to kill the cancer cells. We thus chose IL-7–CBD as the combination agent, because we hypothesized that IL-7 might limit T cell exhaustion. In-depth characterization of the phenotype of tumor-resident T cells revealed that, when compared with the CBD–IL-12 monotherapy, the dual therapy can regulate the immune cell fitness and lead to the decrease of terminally exhausted Tox^high^ Lag3^high^ Tim3^high^ CD8^+^ T cell (*32*, *44*, *45*) population. Instead, the dual therapy expanded the fraction of KLRG1^high^ PD-1^high^ CD8^+^ T cells, which maintain a high tumor-killing potential (*46*). Another noteworthy feature of the dual therapy is that the amount of certain inflammatory markers produced by T cells, such as IFN-γ, was elevated in the tumor but not in the blood when compared to the CBD–IL-12 monotherapy. Given that the dual combination skews the tumor-resident T cells from an exhausted state to a more functional state, we hypothesize that these T cells are able to sustain a high secretion level of IFN-γ for extended period of time.

In addition, treatment of the primary tumor with IL-7–CBD and CBD–IL-12 exhibited notable benefits when compared to the CBD–IL-12 monotherapy in long-term anticancer immune response. The mice cured by the combination therapy showed that circulating CD8^+^ T cells specific for the B16F10 antigen TRP2 were significantly expanded after B16F10 rechallenge, consistent with the known role of IL-7 as a key player in the development of long-term immunity. These mice also had more TRP2-specific CD62L^+^CD44^+^CD8^+^ T cells in the primary tumor-draining lymph node compared to the CBD–IL-12 monotherapy. Thus, all the mice cured by the combination survived in rechallenge experiments, whereas none of the mice cured by CBD–IL-12 survived the rechallenge.

A lack of additive toxicity is certainly a critical feature of the design of our combination approach. We believe that IL-7 receptor expression by CD8^+^ T cells is critical for antitumor efficacy, whereas minimal expression of it on NK cells prevents additional systemic inflammation (*19*). One of the reasons cytokine immunotherapies induce toxicity is that NK cells quickly respond to agents such as IL-2, IL-12, and IL-15 (*47*–*50*) and start secreting other proinflammatory cytokines that lead to organ damage. We also have previously shown that NK cells are the main contributors to the toxicity in IL-12 monotherapy (*29*). However, because murine NK cells have a minimal expression level of the IL-7 receptor and low responsiveness to IL-7 (fig. S23), IL-7–CBD does not significantly contribute to NK cell activation and thus systemic inflammation. Therefore, the wide dose range of IL-7–CBD (5, 10, and 20 μg) combined with CBD–IL-12 (2 μg) did not induce dose-dependent systemic toxicity and increased therapeutic efficacy similarly as the 10-μg CBD–IL-12 monotherapy. This further corroborates the rational choice of IL-7 as the partner cytokine to IL-12. Conversely, IL-15, another common γc family member and representative candidate for combination therapy with IL-12, significantly induced additive toxicity upon the combination with IL-12, likely due to the constitutive expression of its receptor complex on NK cells.

For clinical translation, an important feature of the IL-7–CBD and CBD–IL-12 combination therapy is that synergistic antitumor efficacy is observed upon simultaneous administration of the two agents. In the clinical use, different agents of combination therapies may have different administration schedules due to their respective mechanisms of action and pharmacokinetics, which can increase the burden on medical staff and patients. The simultaneous administration observed to be effective here may be more straightforward and clinically translatable than other described combinations that may require the administration of dual drugs on different schedules (*51*–*53*).

Systemic administration of IL-7–CBD and CBD–IL-12 in conjunction with suboptimal αPD-1 dose showed unprecedented tumor control without any overt toxicity in the autochthonous Braf^V600E^/Pten^−/−^/βCAT^STA^ melanoma model. This genetically engineered mouse model recapitulates many aspects of the CPI-resistant melanoma seen in patients (*41*, *54*, *55*), highlighting the translational potential of our combination therapy.

In summary, we demonstrated that a rational combination of IL-7–CBD, across a wide dose range, with CBD–IL-12 exhibited superior antitumor efficacy by minimizing T cell exhaustion without eliciting additional irAEs and allowed reduction of CBD–IL-12 dose without loss in efficacy. Such therapeutic index enhancement may serve as the basis for designing future combination therapy regimens.

## EXPERIMENTS

### Mice and cancer cell line

Female C57BL/6 mice (age 8 to 12 weeks) were purchased from Charles River Laboratories. Female Balb/C mice (aged 8 to 12 weeks) were purchased from the Jackson Laboratory. B16F10 melanoma and 4T1 breast cancer cell lines were obtained from American Type Culture Collection and were cultured according to instructions. Cell lines were routinely checked for my mycoplasma contamination. Braf^V600E^/Pten^−/−^/βCAT^STA^ mice (aged 8 to 12 weeks) were bred at the animal facility of The University of Chicago. Sample sizes were predetermined from pilot experiments and/or that have been done in the past, to obtain statistically significant data. All mice were randomly mixed before to the start of the in vivo experiments. All the animal experiments performed in this research were approved by the Institutional Animal Care and Use Committee of The University of Chicago (approved protocol number 72470).

### Production and purification of recombinant cytokines

To produce naïve IL-7, sequence-optimized mouse IL-7 was cloned into mammalian expression vector pcDNA3.1(+) by GenScript. A His-tag sequence (His)_6_ was added to the C terminus of IL-7 to enable affinity chromatography. To produce IL-7–CBD, a CBD [A3 domain of von Willebrand factor (VWF) (*22*)]–encoding sequence was inserted into C terminus of IL-7 with a linker (GGGS)_2_, and the His-tag sequence was inserted into C terminus of CBD. Sequence-optimized IL-7–CBD was cloned into mammalian expression vector pcDNA3.1(+) by GenScript. The proteins were expressed in a human embryonic kidney (HEK) 293F system (Invitrogen). Plasmid deoxyribonucleic acid (pDNA) (1 mg/liter) with 25-kDa polyethyleneimine (2 mg/liter; Polysciences) were coincubated in OptiPro SFM medium (4% final volume) for 10 min. The mixture was then transfected into HEK293F cells (1 × 10^6^ cells/ml). After 5 days of the transfection, the supernatant was collected and filtered using 0.2-μm filter unit. The protein purification was performed as described previously (*22*, *24*). Purified proteins were tested for endotoxin using HEK-Blue TLR4 reporter cell line, and endotoxin levels were below 0.01 endotoxin units/ml. Protein purity was confirmed using SDS–polyacrylamide gel electrophoresis as described previously (*22*, *25*). Protein concentration was determined by absorbance method using NanoDrop (Thermo Fisher Scientific). CBD–IL-12 was produced as described previously (*23*). Recombinant mouse IL-15 was purchased from PeproTech Inc. (NJ, USA), and recombinant mouse IL-15R alpha Fc chimera protein was purchased from R&D Systems (MN, USA).

### Analysis of pSTAT5 activity of IL-7 and IL-7–CBD using flow cytometry

Mouse CD3^+^ T cells were purified from the blood of female C57BL/6 mice using an EasySep mouse total (CD3^+^) T cell isolation kit (STEMCELL Technologies). Purified CD3^+^ T cells (1 × 10^6^ cells/ml were rested in cell culture medium for 2 hours and transferred into 96-well plates (50,000 cells per well). The cell culture medium was Iscove's Modified Dulbecco's Medium (IMDM) (Gibco) containing 10% heat-inactivated fetal bovine serum (FBS) and 1% penicillin-streptomycin. Mouse CD3^+^ T cells were stimulated for 20 min at 37°C with various concentrations of IL-7 or IL-7–CBD. Then, cells were permeabilized using BD Phosflow Perm Buffer III for 30 min in 4°C. The cells were stained with Alexa Fluor 647–conjugated phosphorylated signal transducer and activator of transcription 5 (pSTAT5) antibody (clone 47, BD Biosciences) for overnight at 4°C (dilution, 1:50). The cells were acquired using BD LSR flow cytometer, and data were analyzed using FlowJo (TreeStar).

### In vitro collagen I binding affinity of IL-7–CBD

One hundred microliters of human collagen I (10 μg/ml in PBS, EMD Millipore) was coated in medium-binding 96-well plate (Greiner Bio-One) overnight at 37°C. The collagen-coated 96-well was washed three times with PBS-T (1× PBS and 0.05% Tween 20) and blocked with blocking buffer [2% bovine serum albumin (BSA) in PBS-T] at room temperature for 2 hours. Then, the plate was washed three times with PBS-T and added IL-7–CBD or IL-7 at serial dilution in dilution buffer (0.1% BSA in PBS-T) at room temperature for 3 hours. After the incubation, the plate was washed three times with PBS-T and added IL-7 detection antibody (biotinylated) diluted in dilution buffer (dilution factor is 1:100) at room temperature for 1 hour. After the incubation, the plate was washed three times with PBS-T and added avidin–horseradish peroxidase diluted in dilution buffer at room temperature for 30 min. Then, the plate was washed three times with PBS-T and added 100 μl of trimethylboron substrate (EMD Millipore) for colorimetry analysis.

### In vivo evaluation of IL-12 + IL-7 and CBD–IL-12 + IL-7–CBD combination therapies administered intravenously or intratumorally

C57BL/6 mice were inoculated with 5 × 10^5^ B16F10 melanoma cells intradermally on day 0. Tumor-bearing animals were treated with 100 μl of PBS (*n* = 10), 33.3 pmol of CBD–IL-12 (*n* = 10), 33.3 pmol of IL-12 + 666 pmol of IL-7 (*n* = 10), or 33.3 pmol of CBD–IL-12 + 666 pmol of IL-7–CBD (*n* = 10) on days 7, 13, 19, and 25 intravenously. Alternatively, tumor-bearing mice were treated with 30 μl of PBS (*n* = 10), 33.3 pmol of IL-12 + 333 pmol of IL-7 (*n* = 10), or 33.3 pmol of CBD–IL-12 + 333 pmol of IL-7–CBD (*n* = 10) on days 7, 13, 19, and 25 intratumorally. The tumor size was calculated using the following formula: height × width × thickness × (π/6). The mice were euthanized when the tumor size reached 1000 m^3^ and/or based on humane end-point criteria.

### Therapeutic evaluation of IL-7–CBD monotherapy administered intravenously

C57BL/6 mice were inoculated with 5 × 10^5^ MC38 colon cancer cells subcutaneously on day 0 and injected with 100 μl of PBS (*n* = 10), 1.3 nmol of IL-7 (*n* = 10), or 1.3 nmol of IL-7–CBD (*n* = 10) on days 7 and 13. The tumor size was calculated using the following formula: height × width × thickness × (π/6). The mice were euthanized when the tumor size reached 1000 m^3^ and/or based on humane end-point criteria.

### Therapeutic evaluation of high-dose CBD–IL-12 versus CBD–IL-12 + IL-7–CBD administered intravenously or intratumorally

C57BL/6 mice were inoculated with 5 × 10^5^ B16F10 melanoma cells intradermally on day 0 and injected with 100 μl of PBS (*n* = 8), 33.3 pmol of CBD–IL-12 (*n* = 8), 166.5 pmol of CBD–IL-12 (*n* = 8), or 33.3 pmol of CBD–IL-12 + 1.3 nmol of IL-7–CBD (*n* = 8) on day 8. The tumor size was calculated using the following formula: height × width × thickness × (π/6). The mice were euthanized when the tumor size reached 1000 m^3^ and/or based on humane end-point criteria.

To evaluate the effect of lymphocyte egress from the lymph nodes, we injected 25 μg of FTY720 (Selleck Chemical) intraperitoneally daily from days 6 to 25 after B16F10 inoculation.

To study the effects of immunological memory formation, mice that were cured from either a 10-μg CBD–IL-12 monotherapy or a 2-μg CBD–IL-12 + IL-7–CBD combination therapy were rechallenged with 2 × 10^5^ B16F10 tumor cells on day 60 (60 days after the primary tumor inoculation) on the contralateral side of the back.

To understand which immune cell subsets were responsible for the rejection of B16F10 rechallenge, mice that were cured with a 2-μg CBD–IL-12 + IL-7–CBD combination therapy were rechallenged with 2 × 10^5^ B16F10 melanoma cells intradermally on day 60 (60 days after primary tumor inoculation) and injected intraperitoneally with immunoglobulin G (IgG) isotype antibody, 300 μg of CD8 antibody, 300 μg of CD4 antibody, and 300 μg of IFN-γ antibody on days 59, 62, 65, and 68 (after primary tumor inoculation). The following anti-mouse antibodies were used for inhibition: IgG isotype antibody (clone MOPC-21, BioXCell), αCD8 (clone 2.43, BioXCell), αCD4 (clone GK1.5, BioXCell), and αIFN-γ (clone XMG1.2, BioXCell).

### Synergistic effect of CBD–IL-12 combined with IL-7–CBD administered intratumorally

For making B16F10 primary tumor model, C57BL/6 mice were inoculated with 5 × 10^5^ B16F10 melanoma cells intradermally on day 0 and injected with 30 μl of PBS (*n* = 10), 33.3 pmol of CBD–IL-12 (*n* = 10), 333 pmol of IL-7–CBD (*n* = 10), or 33.3 pmol of CBD–IL-12 + 333 pmol of IL-7–CBD (*n* = 10) on day 7. The tumor size was calculated using the following formula: height × width × thickness × (π/6). The mice were euthanized when the tumor size reached 1000 m^3^ and/or based on humane end-point criteria.

### In vivo pharmacokinetic studies of IL-7–CBD + CBD–IL-12 administered intravenously or intratumorally

C57BL/6 mice were inoculated with 5 × 10^5^ B16F10 melanoma cells intradermally on day 0 and injected with 166.5 pmol of IL-12, 166.5 pmol of CBD–IL-12, 666 pmol of IL-7, or 666 pmol of IL-7–CBD intravenously or intratumorally on day 9. The blood was collected at the scheduled time points for the pharmacokinetic studies. The tumors were harvested 24 hours after intravenous injection, and the tumors were harvested 72 hours after intratumoral injection for analysis of the cytokine concentration.

### Blood toxicity testing of high-dose CBD–IL-12 versus CBD–IL-12 + IL-7–CBD administered intravenously

C57BL/6 mice were treated with 100 μl of PBS (*n* = 8), 33.3 pmol of CBD–IL-12 (*n* = 8), 166.5 pmol of CBD–IL-12 (*n* = 8), or 33.3 pmol of CBD–IL-12 + 1.3 nmol of IL-7–CBD (*n* = 8) on day 0. Mice were bled on day 2 for the quantification of cytokine or chemokine levels (IFN-γ, IL-6, TNF-α, CXC motif chemokine ligand 9 (CXCL9), and CXCL10) using LEGENDplex (BioLegend), on day 3 for blood chemistry assay (AST, ALT, amylase, and total bilirubin) using the Vet Axcel Blood Chemistry Analyzer (Alfa Wassermann), and on day 4 for circulating blood cell count (white blood cells, red blood cells, and hemoglobin) using a COULTER Ac•T 5diff CP hematology analyzer (Beckman). For IL-15 superagonist experiment, we used the same LEGENDplex kit and blood chemistry analyzer as for other toxicity evaluation experiments (83.3 pmol of IL-12, 376 pmol of IL-15, and 333 pmol of IL-7).

### Evaluation of cytokine and chemokine expression level in tumor

C57BL/6 mice were inoculated with 5 × 10^5^ B16F10 melanoma cells intradermally on day 0 and injected with 30 μl of PBS (*n* = 8), 33.3 pmol of CBD–IL-12 (*n* = 8), 333 pmol of IL-7–CBD, or 33.3 pmol of CBD–IL-12 + 333 pmol of IL-7–CBD (*n* = 8) on day 7. The tumors were harvested and homogenized using T-PER (Thermo Fisher Scientific) with protease inhibitor tablet (Thermo Fisher Scientific) on day 13. The cytokine and chemokine expression levels in the tumor were analyzed using LEGENDplex (BioLegend).

### High-resolution microscopy of tumor-infiltrating lymphocytes in B16F10 melanoma

C57BL/6 mice were inoculated with 5 × 10^5^ B16F10 melanoma cells intradermally on day 0 and injected with 30 μl of PBS (*n* = 8), 33.3 pmol of CBD–IL-12 (*n* = 8), 333 pmol of IL-7–CBD, or 33.3 pmol of CBD–IL-12 + 333 pmol of IL-7–CBD (*n* = 8) on day 7. The tumors were harvested on 13 day and incubated in 2% paraformaldehyde (PFA) for 2 days at 4°C. The fixed tumors were embedded in paraffin and sectioned to a thickness of 5 μm. The slide staining was performed according to the manufacturer’s protocols. Primary antibody dilution was 1:100 and secondary was 1:200. The following anti-mouse antibodies were used for imaging: CD8 Alexa Fluor 647 (clone 53-6.7, Thermo Fisher Scientific), PD-1 Alexa Fluor 555 (clone J43, Novus Biologicals), and LAG-3 Alexa Fluor 594 (clone EPR20294-77, Abcam).

### Analysis of tumor-infiltrating lymphocytes in the B16F10 melanoma

C57BL/6 mice were inoculated with 5 × 10^5^ B16F10 melanoma cells intradermally on day 0 and injected with 30 μl of PBS (*n* = 8), 33.3 pmol of CBD–IL-12 (*n* = 8), 333 pmol of IL-7–CBD, or 33.3 pmol of CBD–IL-12 + 333 pmol of IL-7–CBD (*n* = 8) on day 7. The tumors were harvested and digested on day 13. In brief, the tumors were cut into small pieces and then digested in 1 ml of digest solution [Dulbecco’s modified Eagle’s medium (DMEM) with 2% FBS, collagenase IV (1 mg/ml; Worthington Biochemical), collagenase D (3.3 mg/ml; Sigma-Aldrich), deoxyribonuclease I (20 μg/ml; Worthington Biochemical), and 1.2 mM CaCl_2_] for 60 min at 37°C on a shaker. Then, the tumor mixture was quenched by 5 mM EDTA, and single-cell suspension was prepared using a cell strainer (70 μm). The single cells were resuspended in DMEM with 2% FBS, and cell staining was performed according to the manufacturer’s protocols (00-5523-00, Thermo Fisher Scientific). The following anti-mouse antibodies were used for flow cytometry: CD45 Alexa Fluor (AF)532 (clone 30-F11, Thermo Fisher Scientific), CD3e Brilliant Ultra Violet (BUV)395 (clone 145-2C11, BD Biosciences), CD4 BUV496 (clone GK1.5, BD Biosciences), CD8a BUV805 (clone 53-6.7, BD Biosciences), CD62L BUV737 (clone MEL-14, BD Biosciences), NK-1.1 APC/Fire810 (clone S17061D, BioLegend), CD44 PerCp-Cy5.5 (clone IM7, BioLegend), PD-1 Brilliant Violet (BV)605 (clone 29F.1A12, BioLegend), Lag3 BV421 (clone C9B7W, BioLegend), Tim3 BV480 (clone 5D12, BD Biosciences), ICOS BV650 (clone C398.4A, BioLegend), CD25 BV785 (clone PC61, BioLegend), CD127 AF647 (clone A7R34, BioLegend), KLRG1 APC-Cy7 (clone 2F1/KLRG1, BioLegend), CD69 PE-Dazzle (clone H1.2F3, BioLegend), Foxp3 AF488 (clone MF23, BD Biosciences), Tox PE (clone TXRX10, Thermo Fisher Scientific), TCF1 Pacific Blue (clone C63D9, Cell Signaling Technologies), and Ki67 PE-Cy7 (clone B56, BD Biosciences). Viability was determined using a fixable, amine reactive dye: Live/Dead Blue Fixable Cell Stain Kit (Thermo Fisher Scientific). Cells were acquired using the Cytek Aurora spectral flow cytometer, and data analysis was performed using FlowJo (TreeStar Inc.).

### Flow cytometry clustering

After spectral unmixing and conventional compensation was performed using a combination of single stained cells and compensation beads (catalog no. 01-3333-42, Thermo Fisher Scientific), CD8^+^ T cells were manually gated as shown (fig. S24). Equal sampling of 1800 CD8^+^ T cells per sample was then concatenated together into one file, and all subsequent analysis was performed. Individual samples could be identified from the concatenated file due to unique keyword identifiers added to the individual samples before concatenation. Dimensionality reduction was performed via UMAP, while FlowSom was used for unsupervised clustering. Both UMAP and FlowSom are available as FlowJo plugins and can be freely downloaded from the FlowJo Exchange. Both dimensionality reduction and clustering were performed using the following markers: CD44, CD62L, Lag3, PD-1, Tim3, TCF1, Tox, CD69, CD127, CD25, ICOS, Ki67, and KLRG1. UMAP was used to visualize the high-dimensional data into one two-dimensional space. Unsupervised clustering was performed using FlowSom. The number of clusters was determined automatically by the algorithm, but the quality of the clustering was also visually confirmed by overlaying the FlowSom clusters on the UMAP. Thus, no modifications to the FlowSom default program were necessary for these data.

### In vitro pSTAT5 activity and IL-7 receptor (CD127) expression in mouse NK cells or CD8^+^ T cells

Mouse NK cells or CD8^+^ T cells were purified from the spleen of female C57BL/6 mice using an EasySep mouse CD8^+^ T cell or NK cell isolation kit (STEMCELL Technologies). Purified mouse cells (1 × 10^6^ cells/ml) were rested in cell culture medium for 2 hours and transferred into 96-well plates (50,000 cells per well). The cell culture medium was RPMI 1640 (Gibco) containing 10% heat-inactivated FBS and 1% penicillin-streptomycin. Mouse cells were stimulated for 20 min at 37°C with various concentrations of IL-7. Then, cells were permeabilized using BD Phosflow Perm Buffer III for 30 min in 4°C. The cells were conducted intracellular staining with pSTAT5 AF647 (clone 47, BD Biosciences) (dilution, 1:50) or cell membrane staining with CD8a BUV805 (clone 53-6.7, BD Biosciences), NK-1.1 APC/Fire810 (clone S17061D, BioLegend), and CD127 AF647 (clone A7R34, BioLegend) (dilution, 1:200) for overnight at 4°C. The cells were acquired using BD LSR flow cytometer, and data were analyzed using FlowJo (TreeStar).

### Inhibitory effect of pulmonary metastatic melanoma

C57BL/6 mice were inoculated with 5 × 10^5^ B16F10 melanoma cells intradermally on day 0. The tumor-bearing mice were administrated 2 × 10^5^ B16F10 melanoma cells intravenously on day 7 and injected with 33.3 pmol of CBD–IL-12 (*n* = 10) or 33.3 pmol of CBD–IL-12 + 666 pmol of IL-7–CBD (*n* = 10) on days 7, 13, and 19. The lungs were harvested on day 25 for measuring the lung weight.

### Histological analysis of IL-12 + IL-7 versus CBD–IL-12 + IL-7–CBD combination therapies

C57BL/6 mice were injected with 100 μl of PBS, 33.3 pmol of IL-12 + 333 pmol of IL-7, or 33.3 pmol of CBD–IL-12 + 333 pmol of IL-7–CBD on day 0 intravenously. Mice were bled days 0, 2, and 4 for blood toxicity assay. The major organs (heart, liver, lung, spleen, and kidney) and tumor were harvested on day 4 for histological analysis. The harvested tissues were incubated in 2% PFA for 2 days at 4°C. The fixed tumors were embedded in paraffin and sectioned to a thickness of 5 μm. H&E and CD8^+^ T cell staining were performed at the Human Tissue Resource Center of The University of Chicago. CD8^+^ T cell–stained area in the liver was analyzed using ImageJ.

### Melanoma antigen (TRP2)–specific immune cell population

Mice whose B16F10 melanoma tumors were cured by either a 166.5-pmol CBD–IL-12 monotherapy (10-μg CBD–IL-12 monotherapy) or a 33.3-pmol CBD–IL-12 + 333-pmol IL-7–CBD combination therapy were rechallenged with 2 × 10^5^ B16F10 melanoma cells intradermally on day 0. Mice were bled on days 0, 3, and 7 after the rechallenge. Red blood cells were lysed by an

ammonium-chloride-potassium (ACK) buffer (Gibco). The single cells were resuspended in DMEM with 2% FBS, and cell staining was performed according to the manufacturer’s protocols (00-5523-00, Thermo Fisher Scientific). BD Horizon Brilliant Stain Buffer was used as a staining buffer. The following anti-mouse antibodies and other reagents were used for flow cytometry: CD45 APC-Cy7 (clone 30-F11, BioLegend), CD3ɛ FITC (clone 500A2, BioLegend), CD4 BV605 (clone GK1.5, BioLegend), CD8 BV421 (clone 53-6.7, BioLegend), CD44 PerCp-Cy5.5 (clone 1 M7, Thermo Fisher Scientific), CD62L PE-Cy7 (clone MEL-14, BioLegend), PD-1 BV711 (clone 29F.1A12, BioLegend), TRP2 pentamer APC (ProImmune), TRP2 pentamer PE (ProImmune), and Live/Dead aqua blue (Thermo Fisher Scientific). Cells were acquired using the BD LSR flow cytometer, and data analysis was performed using FlowJo (TreeStar Inc.).

### Therapeutic evaluation of CBD–IL-12 + IL-7–CBD combined with CPI in 4T1 breast cancer model

Balb/C mice were inoculated with 5 × 10^5^ 4T1 breast cancer cells into the mammary fat pad on day 0 and injected with 100 μg of αPD-1, 83.3 pmol of CBD–IL-12, 666 pmol of IL-7–CBD, or 83.3 pmol of CBD–IL-12 + 666 pmol of IL-7–CBD on days 7, 13, 19, and 25.

### Therapeutic evaluation of CBD–IL-12 + IL-7–CBD combined with CPI in Braf^V600E^/Pten^−/−^/βCAT^STA^ melanoma model

Braf^V600E^/Pten^−/−^/βCAT^STA^ mice were intratumorally treated with 25 μg of tamoxifen on day 0 and injected intravenously with 100 μg of αPD-1, 83.3 pmol of CBD–IL-12, or 83.3 pmol of CBD–IL-12 + 666 pmol of IL-7–CBD on days 25, 32, 39, 46, and 53. Tumor size and body weight measurement were conducted twice a week, and photograph of the tumor was taken on day 53.
