# Supplementary material for: Engineered IL-7 synergizes with IL-12 immunotherapy to prevent T cell exhaustion and promote memory without exacerbating toxicity
Source: Sci Adv. 2023 Nov 29;9(48):eadh9879. doi: 10.1126/sciadv.adh9879 (PMC10686557; doi:10.1126/sciadv.adh9879)
Supplement: Supplementary file 1 — Figs. S1 to S24 [file sciadv.adh9879_sm.pdf]

Science Advances
AAAS

Supplementary Materials for

Engineered IL-7 synergizes with IL-12 immunotherapy to prevent T cell exhaustion and promote memory without exacerbating toxicity

Seounghun Kang *et al.*

Corresponding author: Jeffrey A. Hubbell, jhubbell@uchicago.edu

Sci. Adv. **9**, eadh9879 (2023)
DOI: 10.1126/sciadv.adh9879

This PDF file includes:

Figs. S1 to S24

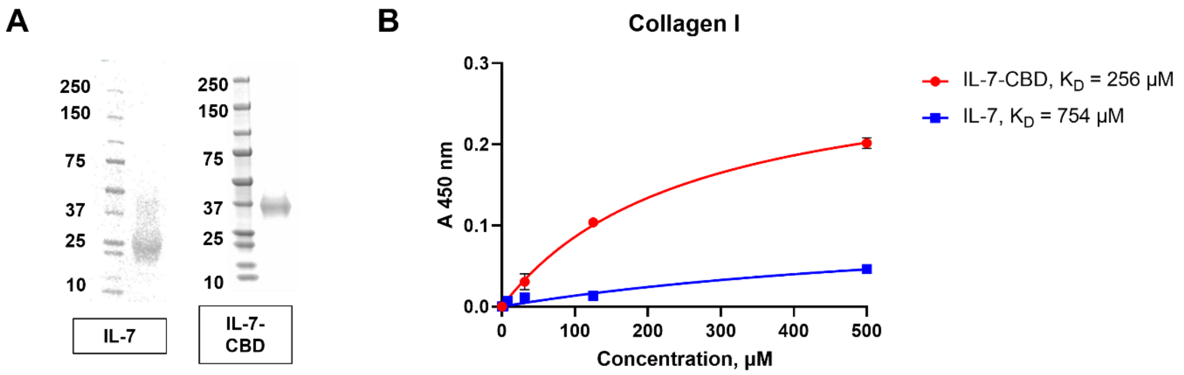

Supplementary Fig. 1. Biophysical characterization of IL-7 and IL-7-CBD. A. SDS-PAGE for IL-7 and IL-7-CBD under a non-reducing condition. As expected, IL-7 shows ~16.0 kDa and IL-7-CBD shows 37.1 kDa size. **B.** Collagen-binding affinity of IL-7-CBD to collagen I.

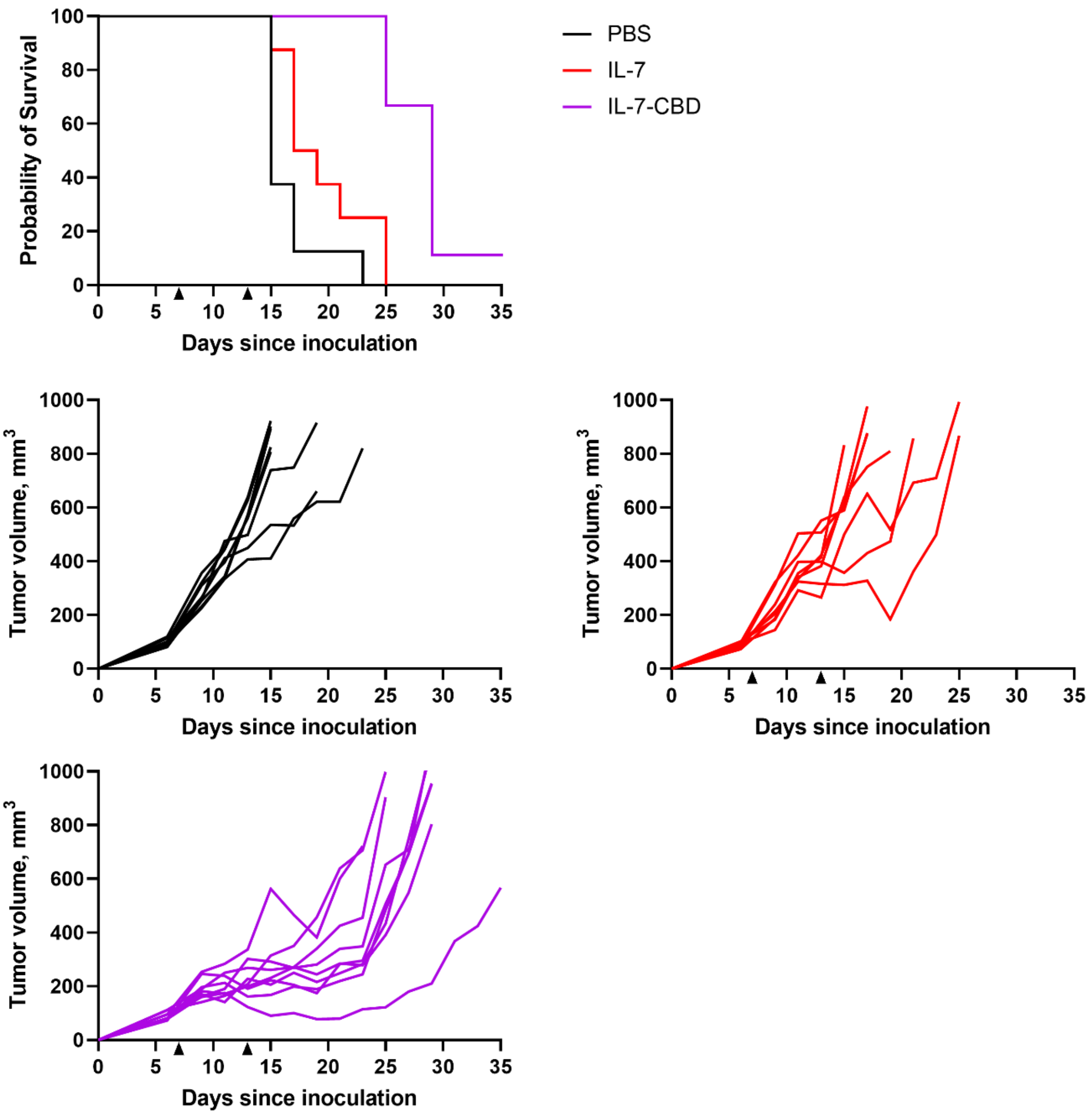

Supplementary Fig. 2. Therapeutic evaluation of intravenously administered IL-7-CBD monotherapy in MC38 model. C57BL/6 mice were inoculated with 5 x 10^5 MC38 cancer cells subcutaneously and injected with 100 μL PBS (n=10), 1.3 nmol IL-7 (i.v., n=10), or 1.3 nmol IL-7-CBD (i.v., n=10) on day 7 and 13.

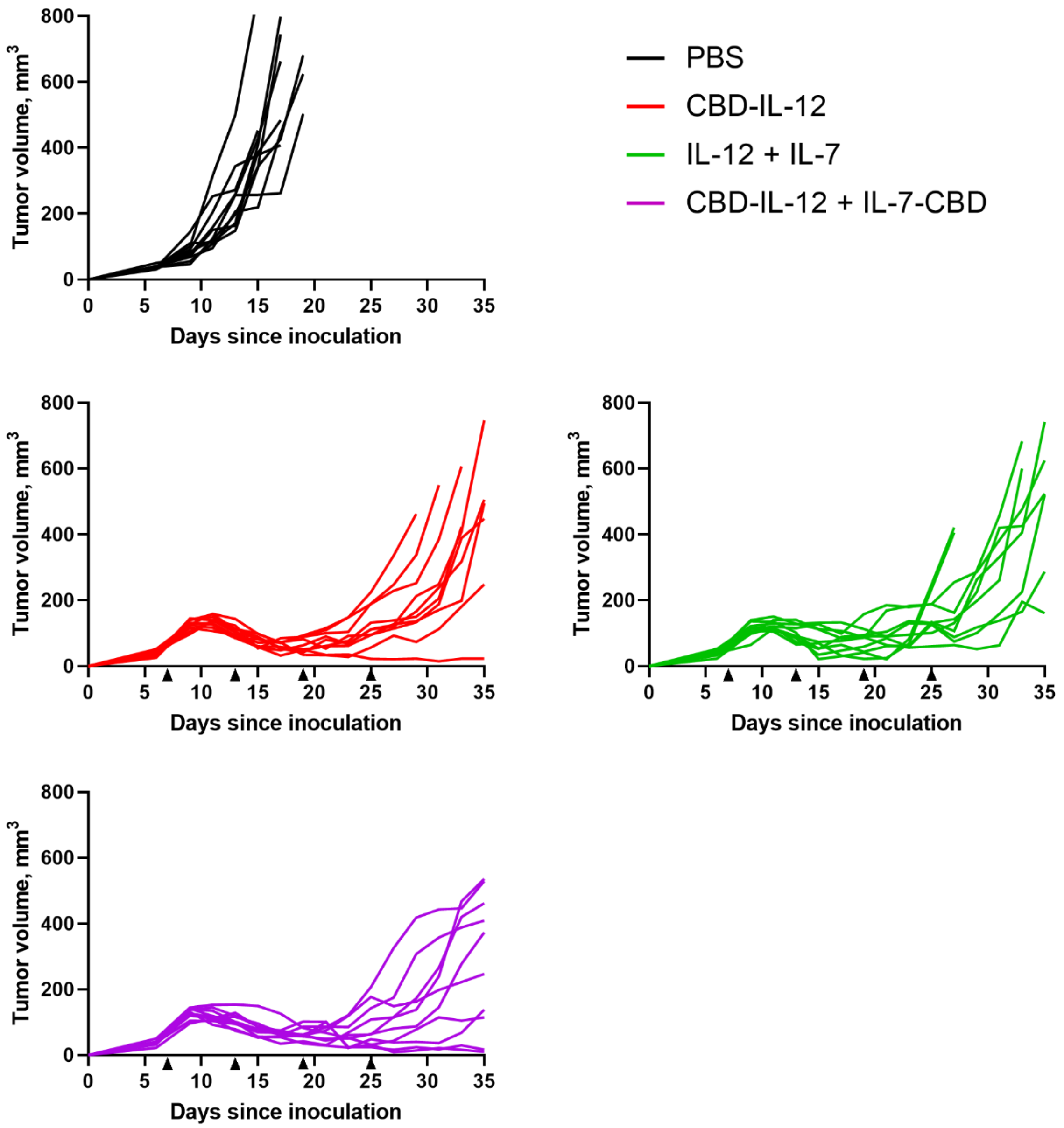

Supplementary Fig. 3. Intravenously administered CBD-fused cytokines show a stronger overall antitumor effect than unmodified cytokines in B16F10 model. C57BL/6 mice were inoculated with 5 x 10^5 B16F10 melanoma intradermally and injected with 30 µL PBS (n=10), 33.3 pmol CBD-IL-12 (i.v., n=10), 33.3 pmol IL-12 (i.v.) + 666 pmol IL-7 (i.v., n=10), or 33.3 pmol CBD-IL-12 (i.v.) + 666 pmol IL-7-CBD (i.v., n=10) on day 7, 13, 19, and 25.

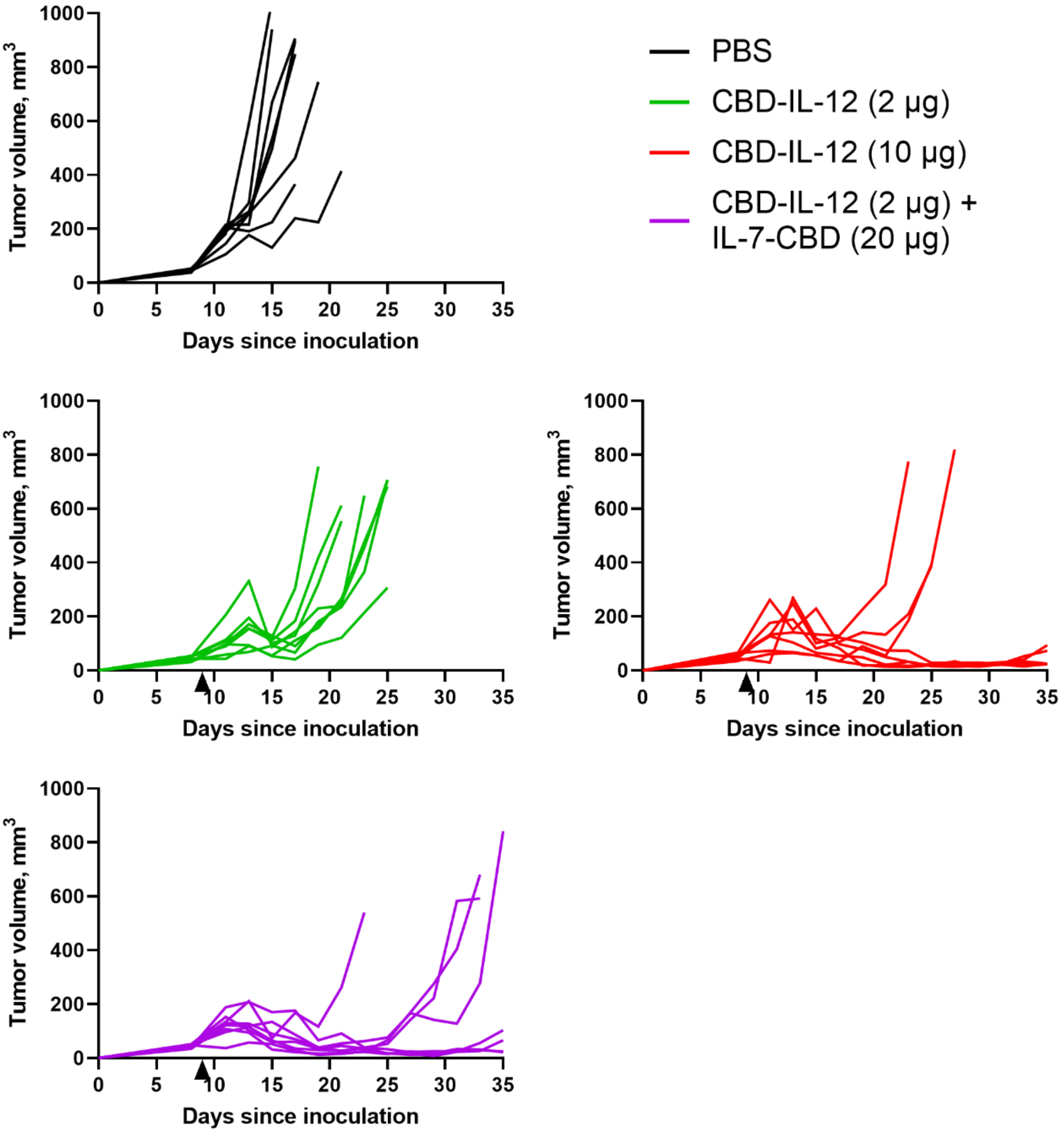

Supplementary Fig. 4. Addition of IL-7-CBD to CBD-IL-12 significantly increases therapeutic scores in B16F10 model. C57BL/6 mice were inoculated with 5 x 10^5 B16F10 melanoma intradermally and injected with 100 µL PBS (n=8), 33.3 pmol CBD-IL-12 (i.v., n=8), 166.5 pmol CBD-IL-12 (i.v., n=8), or 1.3 nmol IL-7-CBD (i.v.) + 33.3 pmol CBD-IL-12 (i.v., n=8) on day 9.

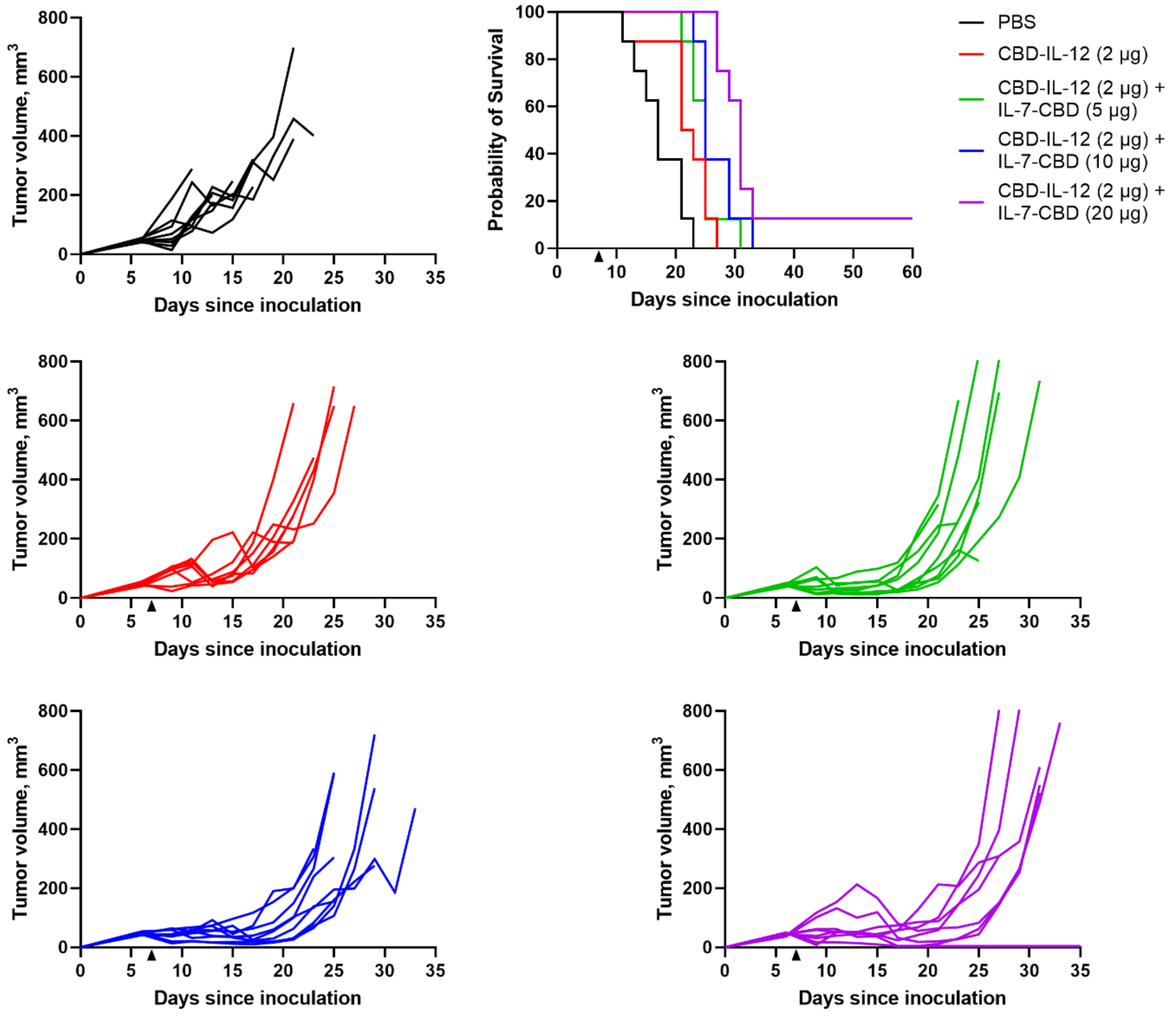

Supplementary Fig. 5. Escalating the dose of IL-7-CBD combined with CBD-IL-12 increased the overall antitumor effect depending on IL-7-CBD dose. C57BL/6 mice were inoculated with 5 x 10^5 B16F10 melanoma intradermally and injected with 100 µL PBS (n=8), 33.3 pmol CBD-IL-12 (i.v., n=8), 333 pmol IL-7-CBD (i.v.) + CBD-IL-12 (i.v., n=8), 666 pmol IL-7-CBD (i.v.) + 33.3 pmol CBD-IL-12 (i.v., n=8), or 1.3 nmol IL-7-CBD (i.v.) + 33.3 pmol CBD-IL-12 (i.v., n=8) on day 7.

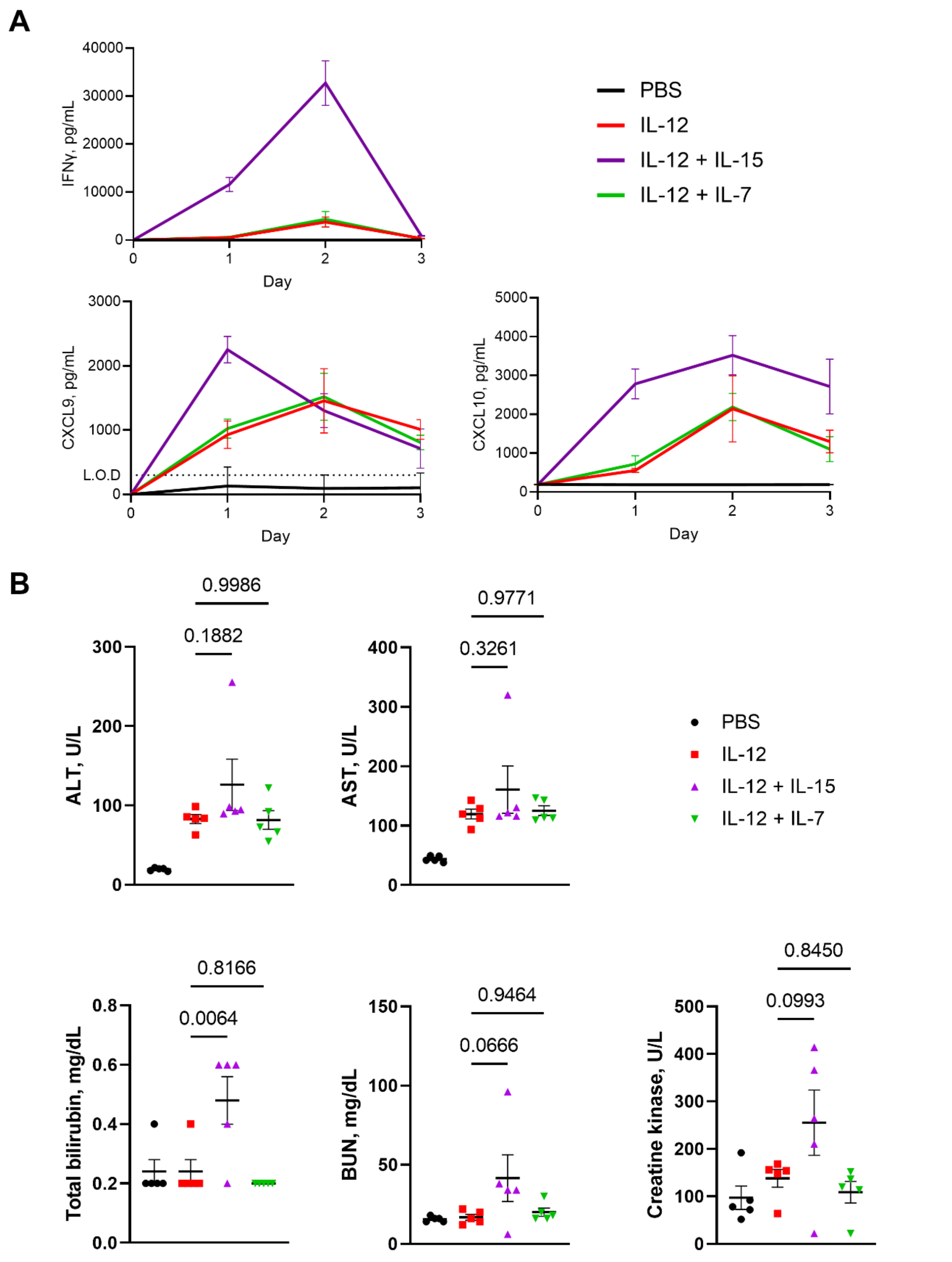

Supplementary Fig. 6. Blood toxicology study of IL-12, IL-15, and IL-7. C57BL/6 mice were injected with 83.3 pmol IL-12 (i.v., n=5), 83.3 pmol IL-12 (i.v.) + 375 pmol IL-15 superagonist (i.v., n=5), or 83.3 pmol IL-12 (i.v.) +333 pmol IL-7 (i.v., n=5) and the mice blood was collected on the scheduled timepoints for toxicity tests. **A.** Systemic inflammation cytokines or chemokines (IFNγ, CXCL9, and CXCL10) expression level and **B.** Blood chemistry assay (ALT, AST, Total bilirubin, BUN, and Creatine kinase) on day 3. Statistical analyses were performed using one-way ANOVA tests or t-test.

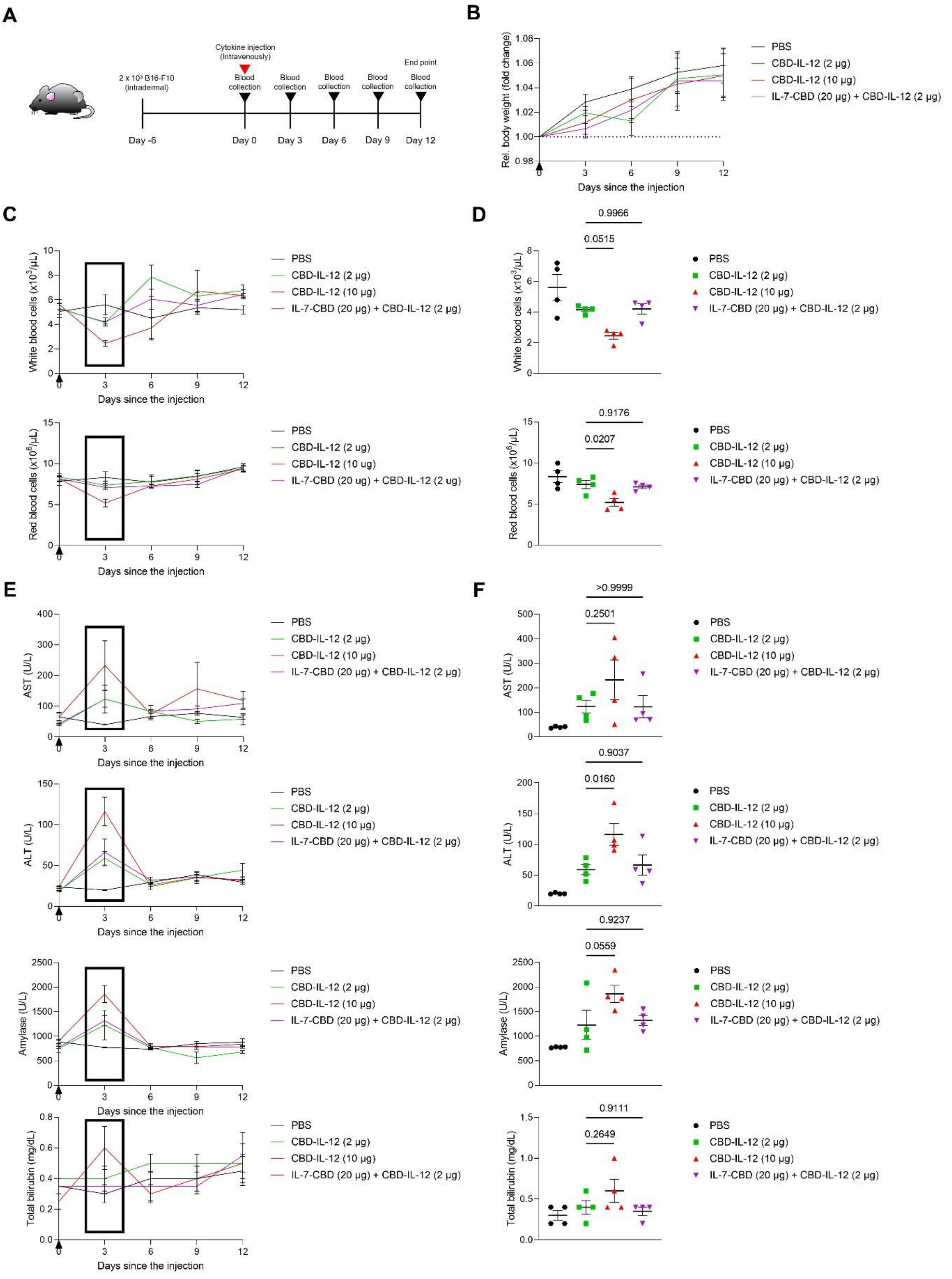

Supplementary Fig. 7. Blood toxicology study of single-dose injection in B16F10-bearing mice. B16F10-bearing C57BL/6 mice were injected with PBS (i.v., n=4), 33.3 pmol CBD-IL-12 (i.v., n=4), 166.5 pmol CBD-IL-12 (i.v., n=4), or 1.3 nmol IL-7-CBD + 33.3 pmol CBD-IL-12 (i.v., n=4), and the blood was collected for the blood cell counts or blood chemistry analysis. **A.** Designed experiment schedule. **B.** Body weight was measured during the experiment schedule. **C.** Blood cell counts and **D.**

Statistical analysis on day 3. **E.** Blood chemistry analysis and **F.** Statistical analysis on day 3. Statistical analyses were performed using one-way ANOVA tests or t-tests. (*: $0.05>P$, **: $0.01>P$, and ***:$0.001>P$)

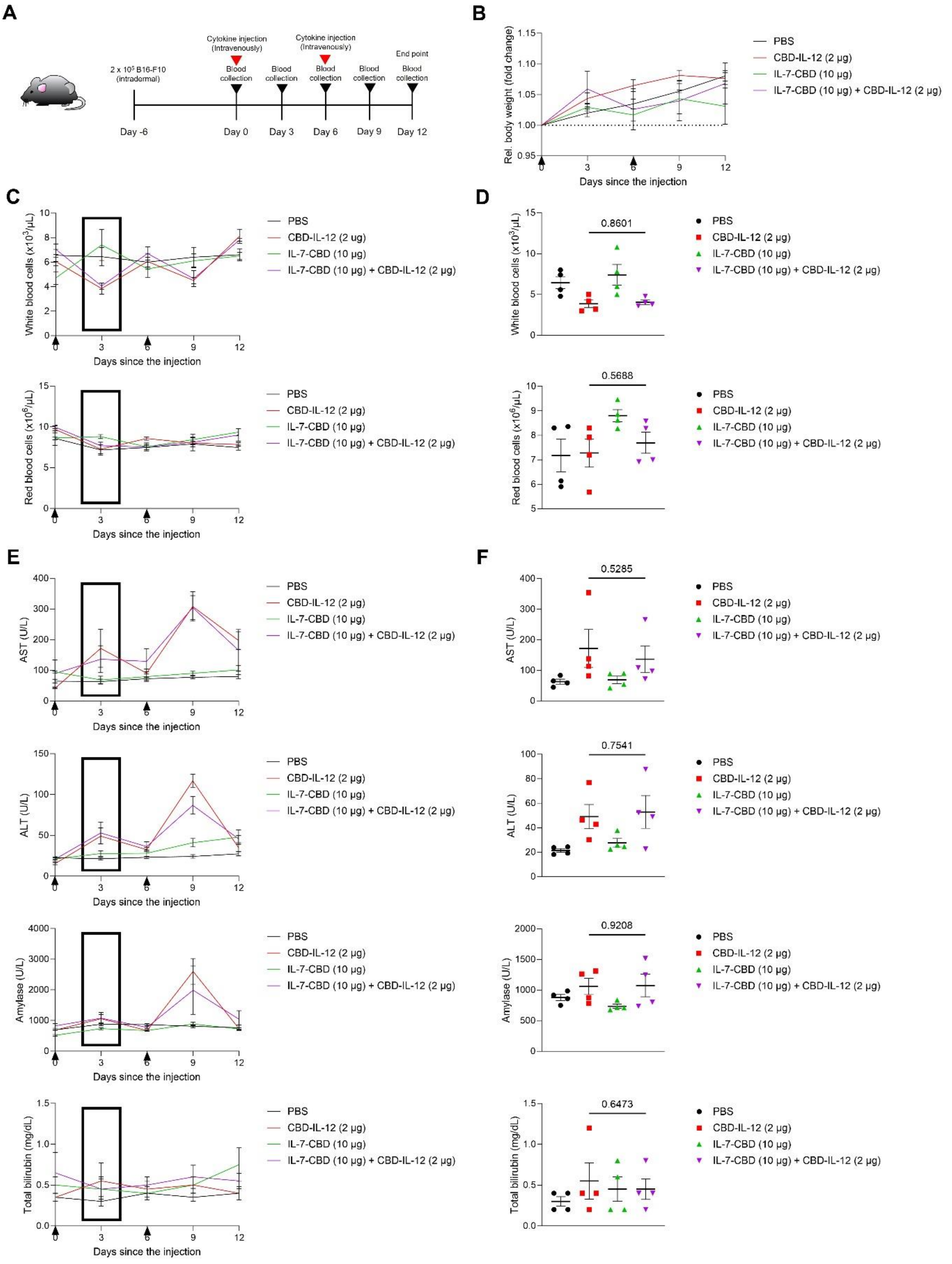

Supplementary Fig. 8. Blood toxicology study of multiple(dual)-dose injection in B16F10-bearing mice. B16F10-bearing C57BL/6 mice were injected with PBS (i.v., n=4), 33.3 pmol CBD-IL-12 (i.v., n=4), 666 pmol IL-7-CBD (i.v., n=4), 666 pmol IL-7-CBD + 33.3 pmol CBD-IL-12 (i.v., n=4) on day 0 and 6, and the blood was collected for the blood cell counts or blood chemistry analysis. **A.** Designed experiment schedule. **B.** Body weight was measured during the experiment schedule. **C.** Blood cell

counts and **D.** Statistical analysis on day 3. **E.** Blood chemistry analysis and **F.** Statistical analysis on day 3. Statistical analyses were performed using one-way ANOVA tests or t-tests. (*: $0.05>P$, **: $0.01>P$, and ***: $0.001>P$)

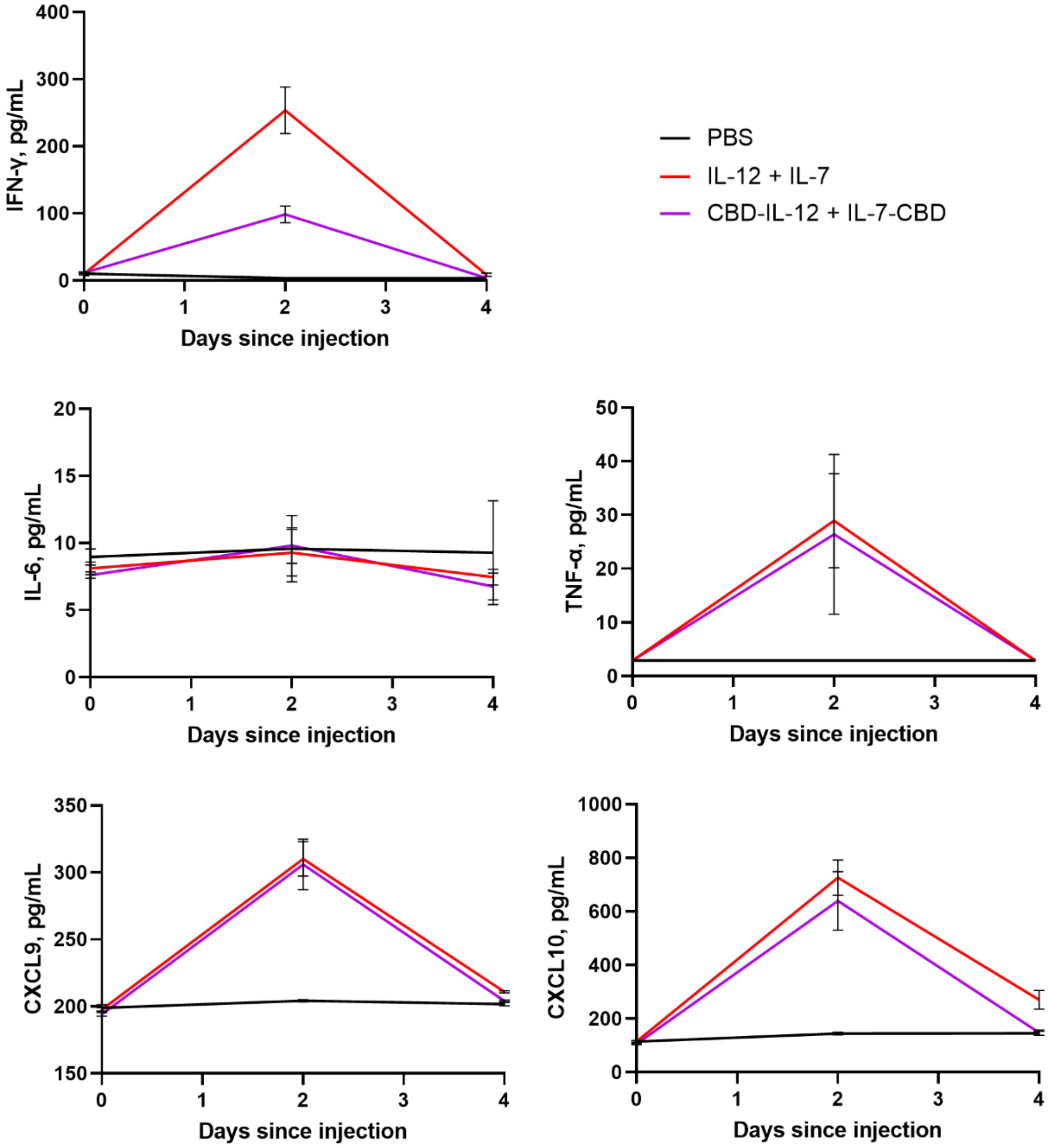

Supplementary Fig. 9. Blood inflammatory cytokine and chemokine expression levels simulated by CBD-fused cytokines or unmodified cytokines. C57BL/6 mice were injected with 100 µL PBS (i.v., n=5), 33.3 pmol IL-12 (i.v.) + 666 pmol IL-7 (i.v., n=5), or 33.3 pmol CBD-IL-12 (i.v.) + 666 pmol IL-7-CBD (i.v., n=5) and the mice blood was collected on the scheduled timepoints for the analysis of inflammatory molecules such as IFNγ, IL-6, TNF-α, CXCL9, and CXCL10.

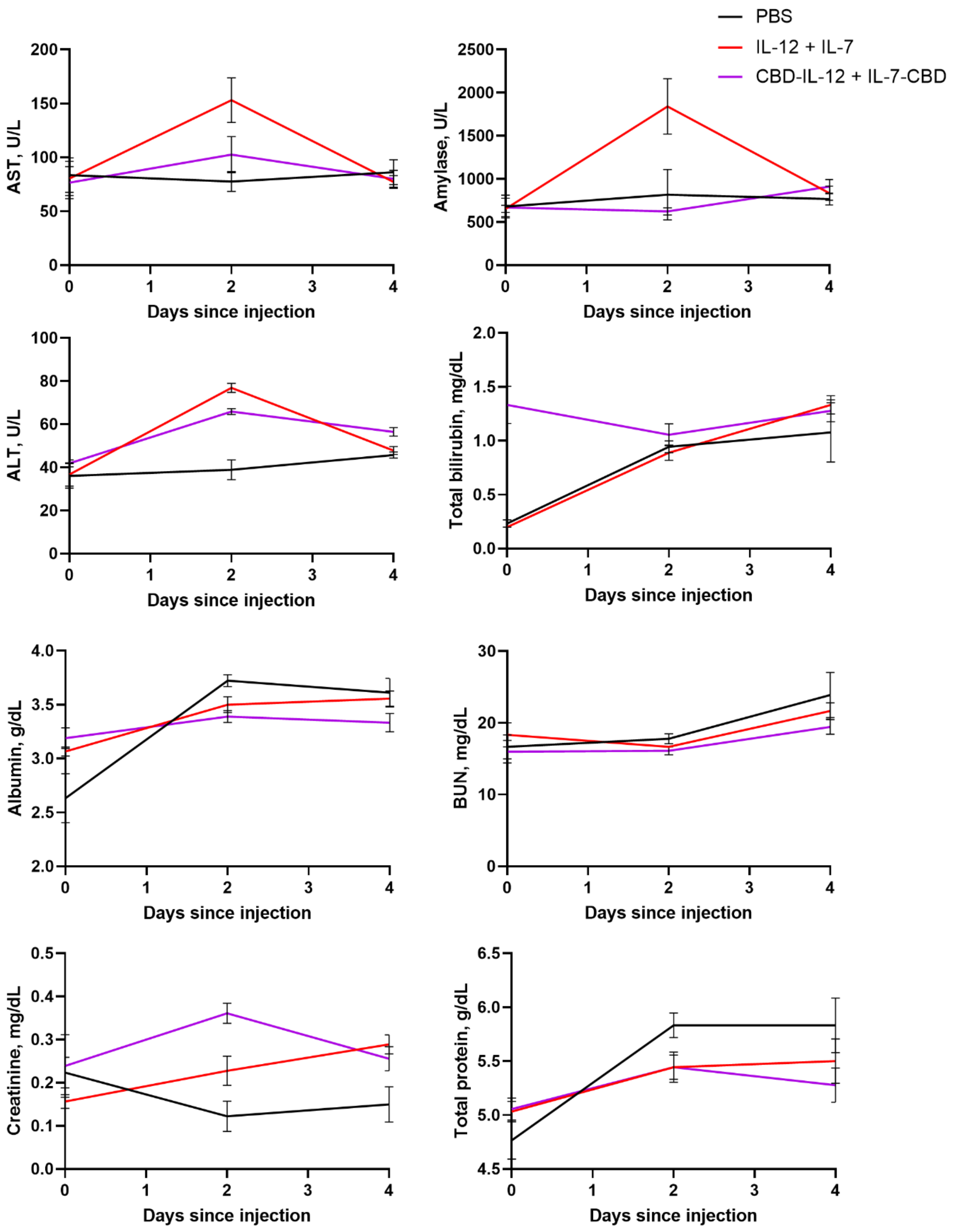

Supplementary Fig. 10. Systemic toxicity induced by CBD-fused cytokines or unmodified cytokines. C57BL/6 mice were injected with 100 µL PBS (i.v., n=5), 33.3 pmol IL-12 (i.v.) + 666 pmol IL-7 (i.v., n=5), or 33.3 pmol CBD-IL-12 (i.v.) + 666 pmol IL-7-CBD (i.v., n=5) and the mice blood was collected on the scheduled timepoints for the blood chemistry analysis such as AST, ALT, Amylase, Total bilirubin, albumin, BUN, Creatinine, and Total protein.

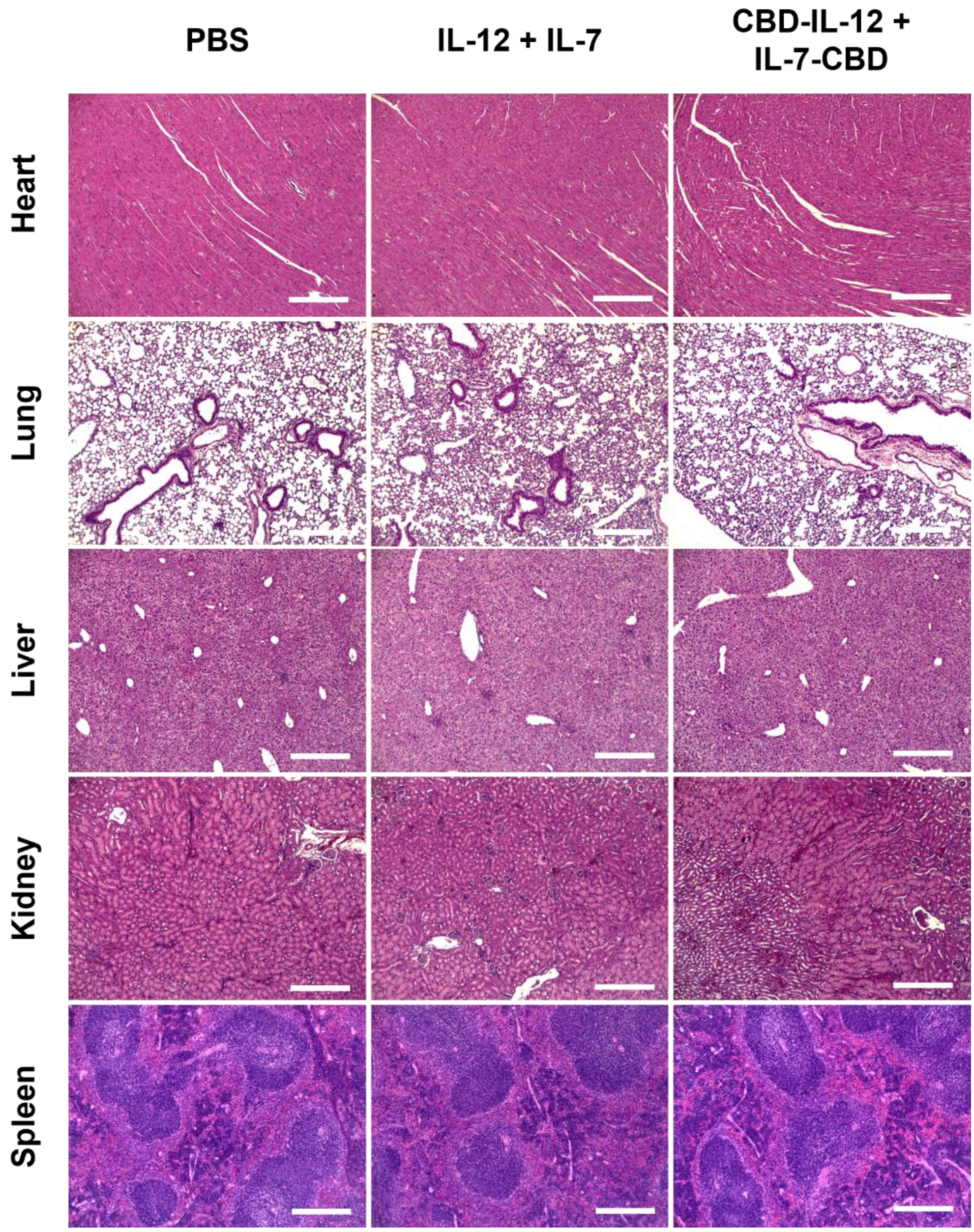

Supplementary Fig. 11. The organ histology in CBD-fused cytokines or unmodified cytokines administered mice. C57BL/6 mice were injected with 100 µL PBS (i.v., n=5), 33.3 pmol IL-12 (i.v.) + 666 pmol IL-7 (i.v., n=5), or 33.3 pmol CBD-IL-12 (i.v.) + 666 pmol IL-7-CBD (i.v., n=5) and the major organs (heart, liver, lung, spleen, and kidney) in the mice were harvested on day 4 for immunohistochemistry of mouse CD8. The organs were incubated in 2% PFA for 2 days at 4 ºC, and the paraffin embedding, section, and H&E staining process were performed at the Human Tissue Resource Center, The University of Chicago. The scale bar is 400 µm.

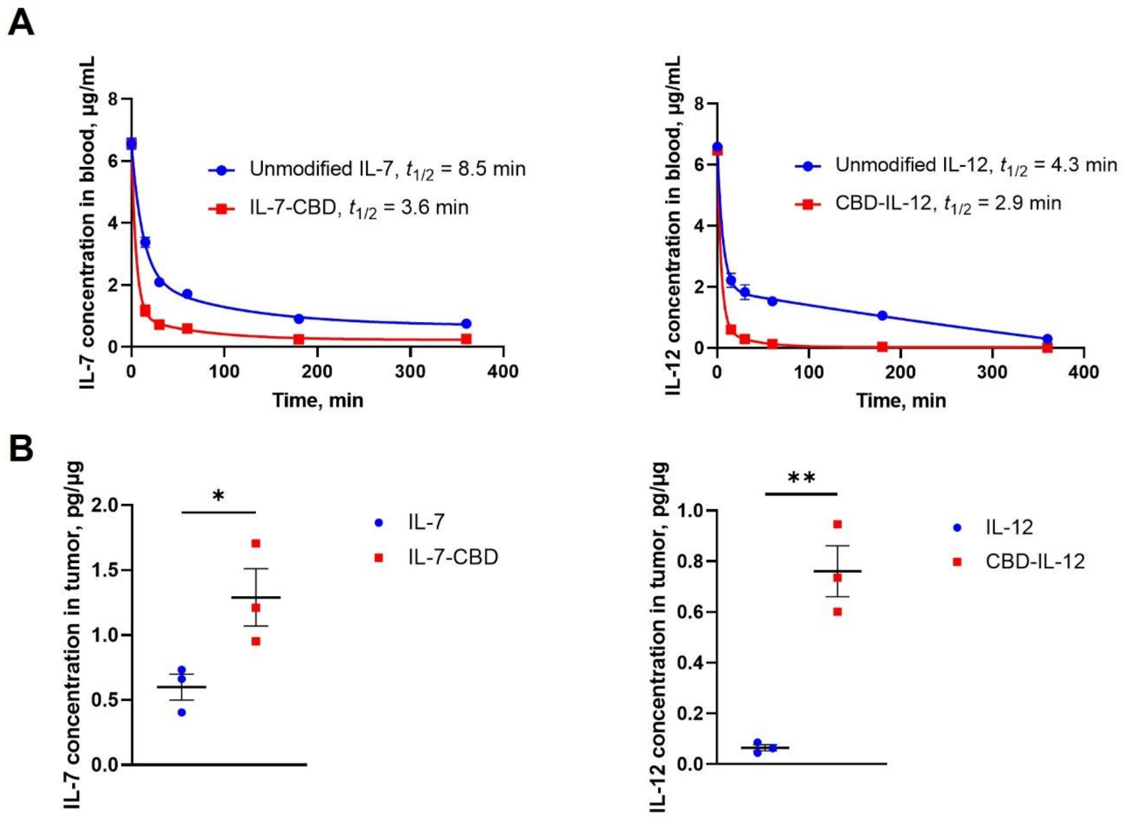

Supplementary Fig. 12. Pharmacokinetic study and tumor accumulation efficacy of IL-7-CBD and CBD-IL-12. B16F10-bearing C57BL/6 mice were injected with 666 nmol IL-7 (i.v., n=3), 666 nmol IL-7-CBD (i.v., n=3), 166.5 pmol IL-12 (i.v., n=3), or 166.5 pmol CBD-IL-12 (i.v., n=3). **A.** The blood was collected at the scheduled time points for the pharmacokinetics study and **B.** The tumors were harvested 24 hr after the injection for analysis of the cytokine concentration. Statistical analyses were performed using one-way ANOVA tests or t-tests. (*: *0.05>P*, **: *0.01>P*, and ***:*0.001>P*)

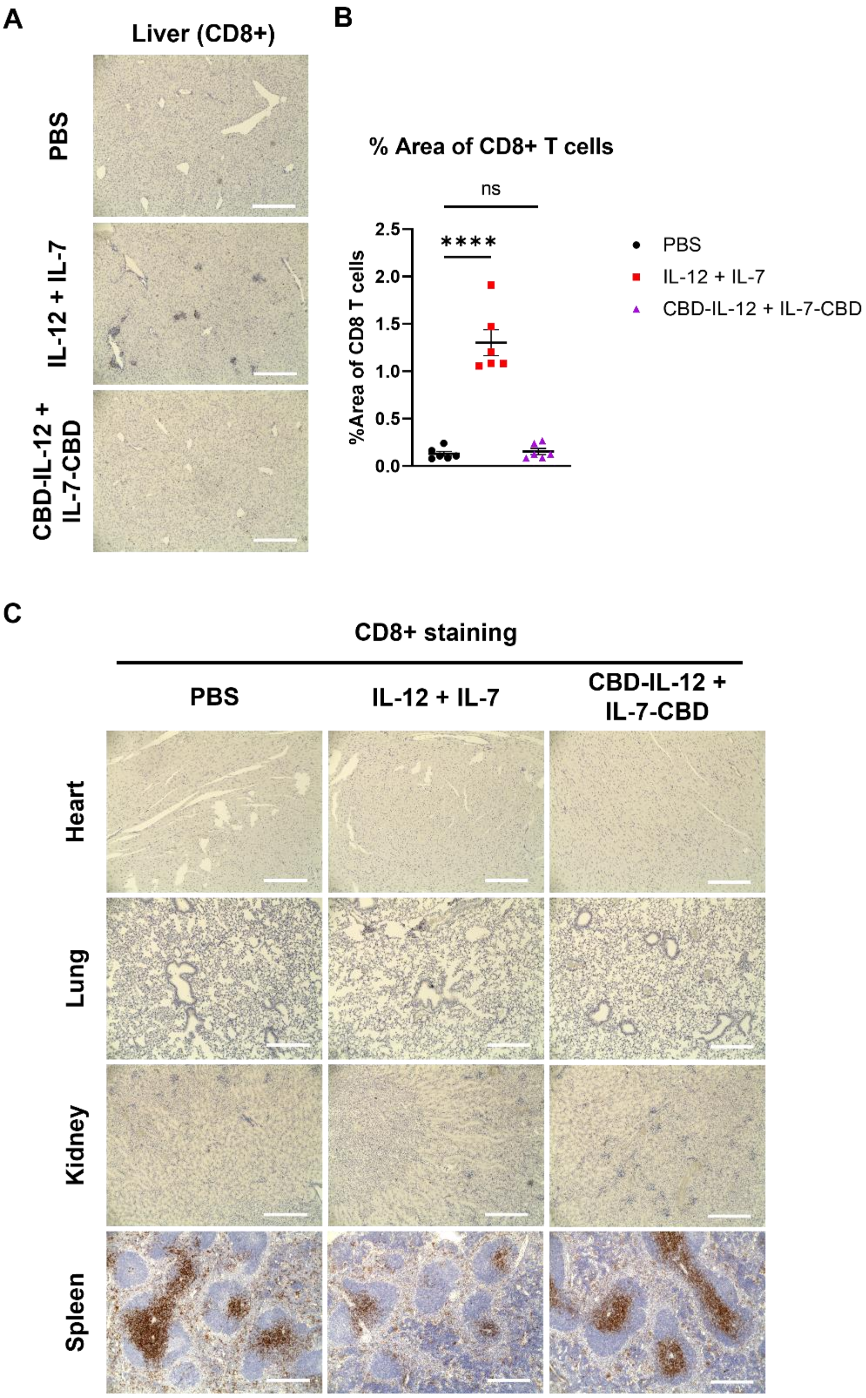

Supplementary Fig. 13. CBD-fused cytokines recruit fewer CD8^{+} T cells into the hepatic region compared to unmodified cytokines. C57BL/6 mice were injected with 100 µL PBS (i.v., n=5), 33.3 pmol IL-12 (i.v.) + 666 pmol IL-7 (i.v., n=5), or 33.3 pmol CBD-IL-12 (i.v.) + 666 pmol IL-7-CBD (i.v., n=5) and the major organs (heart, liver, lung, spleen, and kidney) in the mice were harvested on day 4 for immunohistochemistry of mouse CD8. The organs were incubated in 2% PFA for 2 days at 4 ºC, and the paraffin embedding, section, and CD8 staining process were performed at the Human Tissue Resource Center, The University of Chicago. **A.** Representative images of hepatic recruited CD8+ T cells and **B.** Quantification data. **C.** CD8 staining images of heart, lung, kidney, and spleen. The scale bar is 400 µm. Statistical analyses were performed using one-way ANOVA tests. (*: *0.05>P*, **: *0.01>P*, and ***: *0.001>P*)

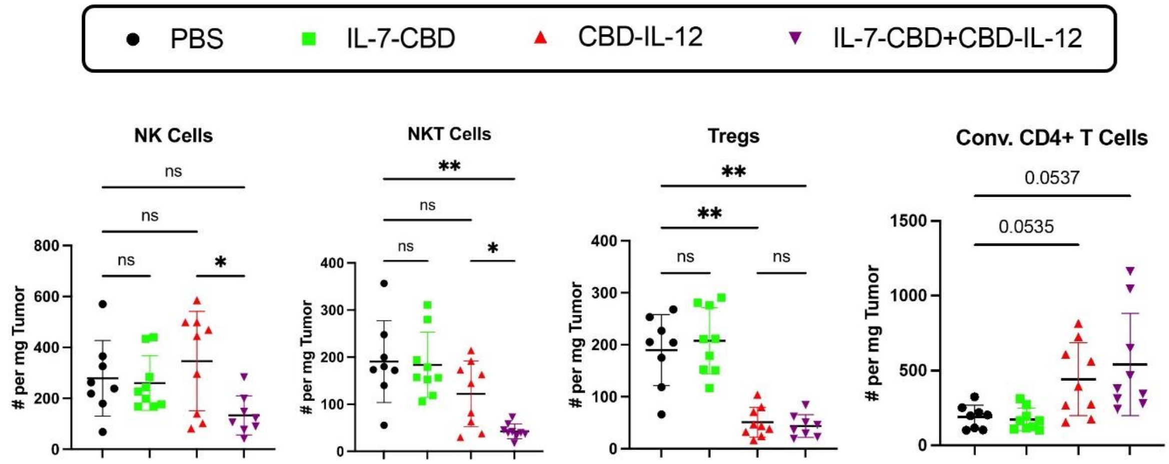

Supplementary Fig. 14. Combination therapy reduces NK and NKT cell numbers compared to CBD-IL-12 monotherapy, but does not alter CD4$^+$ T cell populations compared to CBD-IL-12 alone B16F10 bearing mice were treated i.t. with either PBS, 33.3 pmol CBD-IL-12, 333 pmol IL-7-CBD, or 33.3 pmol CBD-IL-12 + 333 pmol IL-7-CBD, on day 7 and tumors were harvested day 13. Cells were digested into a single cell suspension, stained, and run by flow cytometry. Overall counts of NK cells, NKT cells, Tregs, and conventional CD4$^+$ T cells. Statistical analyses were performed using one-way ANOVA tests. (*: *0.05>P*, **: *0.01>P*, and ***: *0.001>P*)

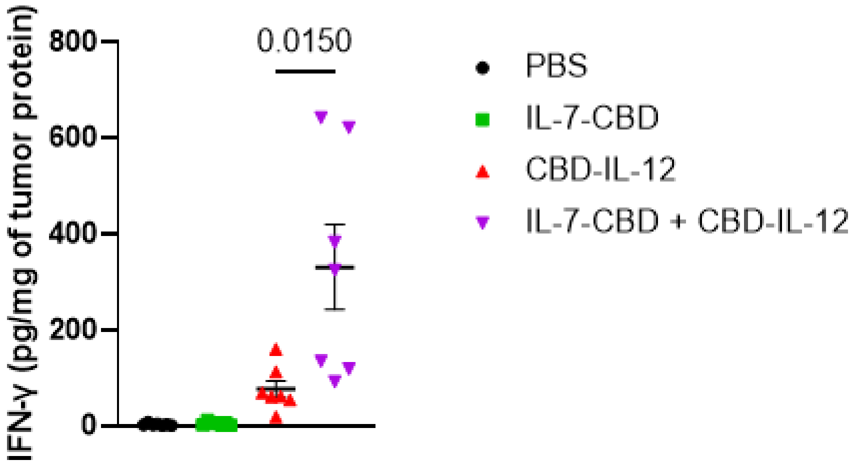

Supplementary Fig. 15. IL-7-CBD + CBD-IL-12 combination therapy significantly stimulated intratumoral IFN-γ compared CBD-IL-12 monotherapy. C57BL/6 mice were inoculated with 5 x 10^5 B16F10 melanoma intradermally and injected with 30 µL PBS (i.t., n=7), 333 pmol IL-7-CBD (i.t., n=7), 33.3 pmol CBD-IL-12 (i.t., n=7), or 333 pmol IL-7-CBD (i.t.) + 33.3 pmol CBD-IL-12 (i.t., n=10) on day 7. The tumors were harvested on day 13 for analysis of cytokine expression level.

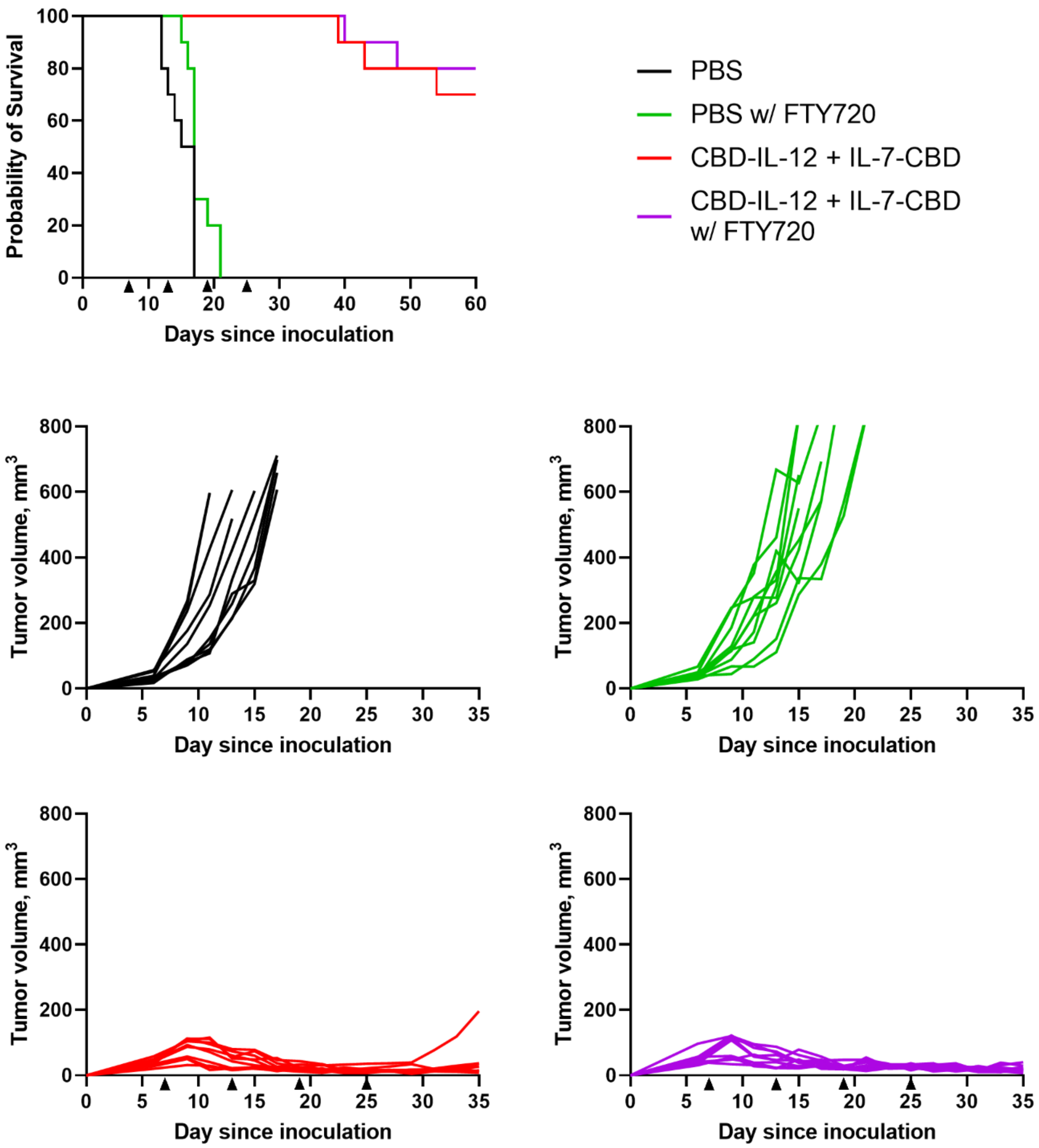

Supplementary Fig. 16. Intratumorally administered combination therapy show sufficient antitumor effect even with only immune cells in the circulating system. C57BL/6 mice were inoculated with 5 x 10^5 B16F10 melanoma intradermally and injected with 30 μL PBS (i.t., n=10) or 333 pmol IL-7-CBD (i.t.) + 33.3 pmol CBD-IL-12 (i.t., n=10) on day 7, 13, 19, and 25. we injected 25 μg FTY720 intraperitoneally daily from days 6 to 25 after B16F10 inoculation.

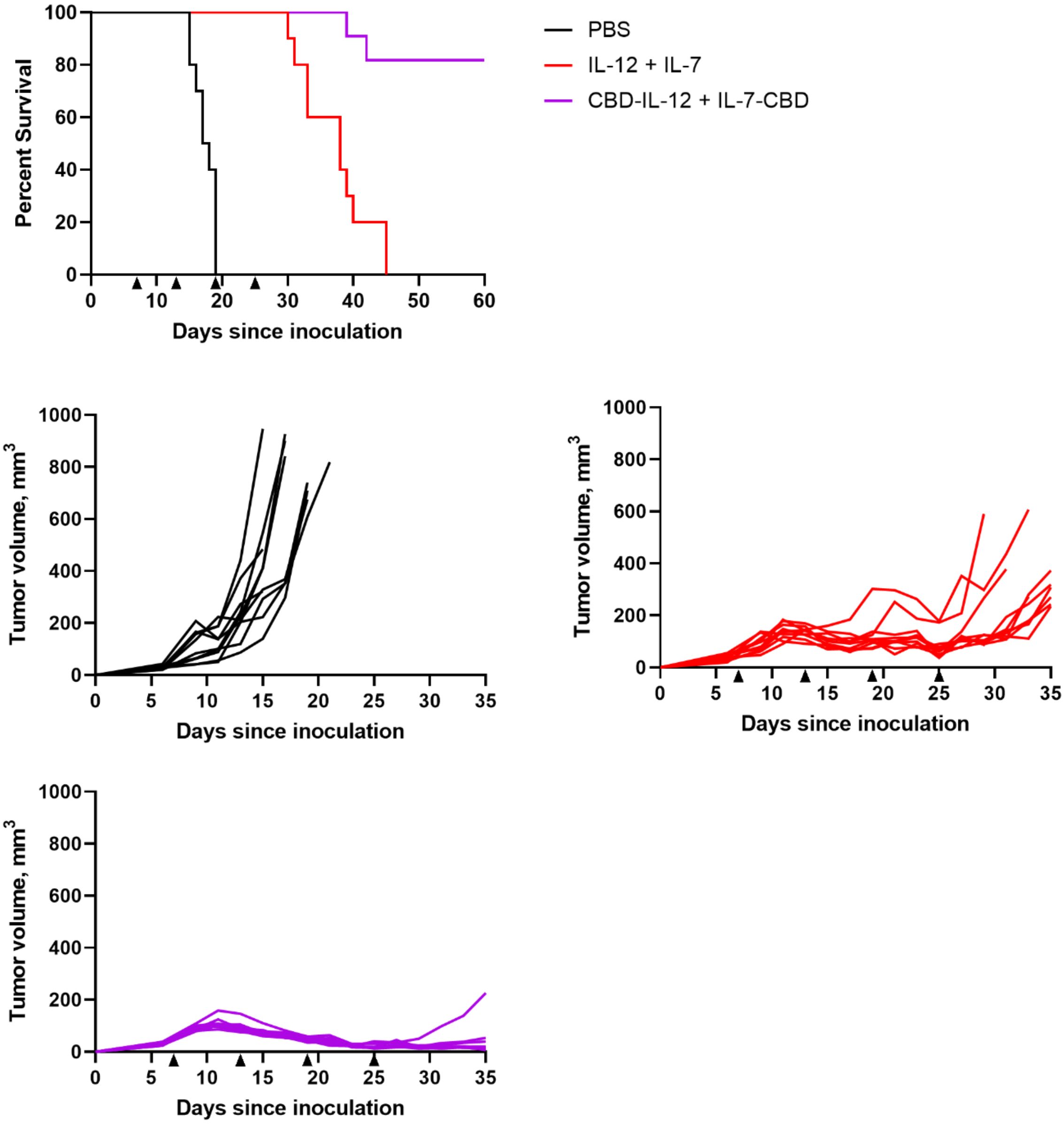

Supplementary Fig. 17. Therapeutic evaluation of intratumorally administered CBD-fused cytokines in B16F10 model. C57BL/6 mice were inoculated with 5 x 10^5 B16F10 melanoma intradermally and injected with 30 µL PBS (i.t., n=10), 33.3 pmol IL-12 (i.t.) + 333 pmol IL-7 (i.t., n=10), or 33.3 pmol CBD-IL-12 (i.t.) + 333 pmol IL-7-CBD (i.t., n=10) on day 7, 13, 19, and 25.

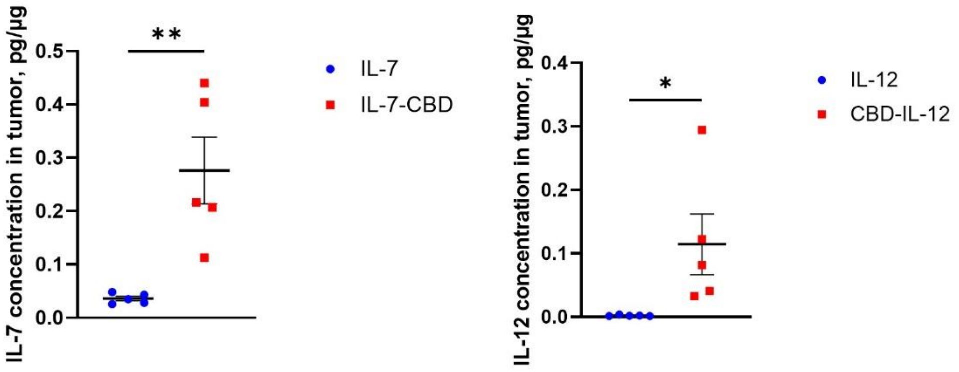

Supplementary Fig. 18. The CBD fusion to IL-7 or IL-12 prolongates the intratumoral retention of the cytokines. B16F10-bearing C57BL/6 mice were injected with 666 nmol IL-7, 666 nmol IL-7-CBD, 166.5 pmol IL-12, or 166.5 pmol CBD-IL-12 intratumorally. The tumors were harvested 72 hr post intratumoral injection of the cytokines for analysis of the cytokine concentration. Statistical analyses were performed using one-way ANOVA tests or t-tests. (*: $0.05>P$, **: $0.01>P$, and ***:$0.001>P$)

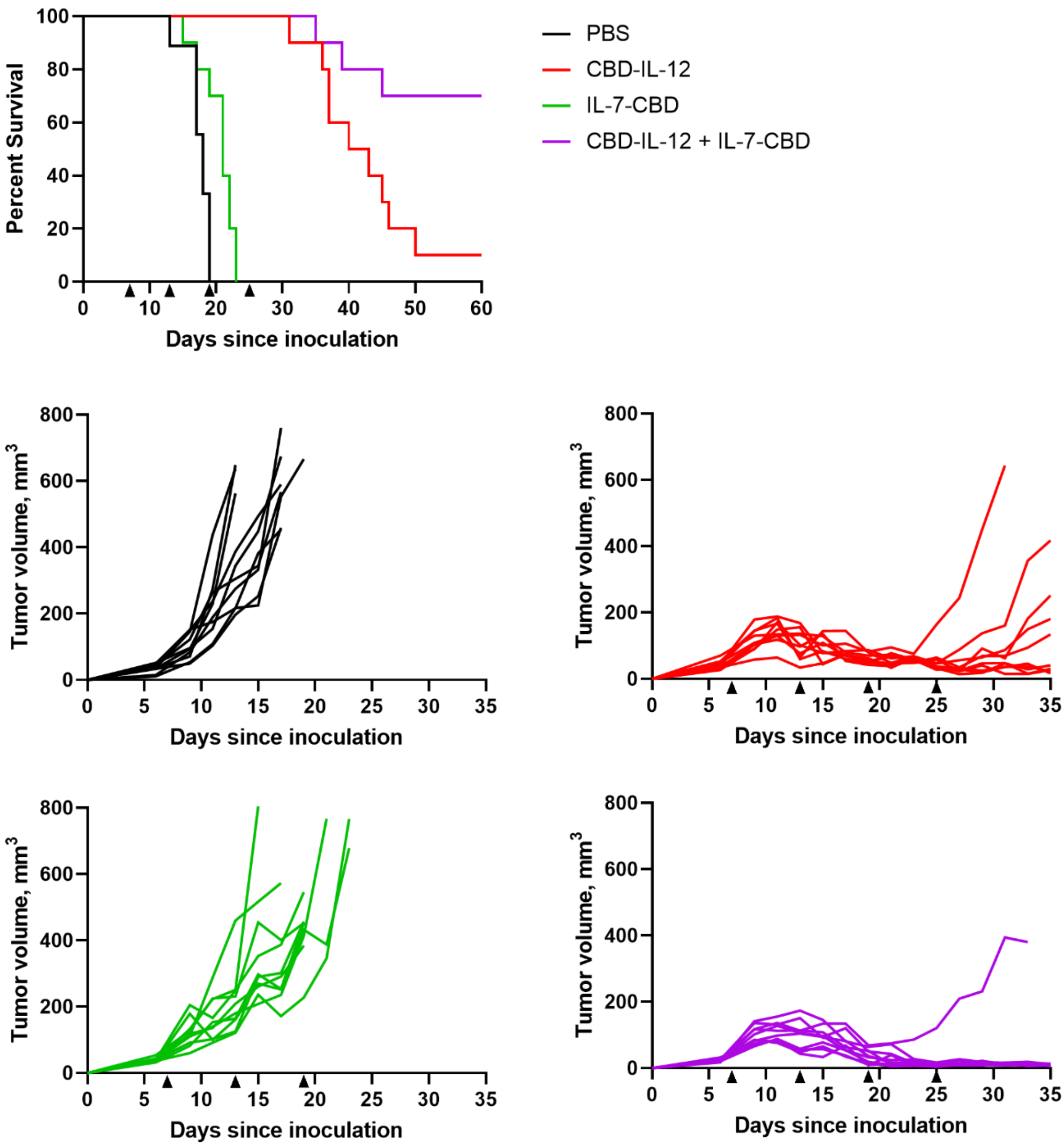

Supplementary Fig. 19. Synergistic effect of intratumorally administered CBD-fused cytokines in B16F10 model. C57BL/6 mice were inoculated with 5 x 10^5 B16F10 melanoma intradermally and injected with 30 μL PBS (i.t., n=10), 33.3 pmol CBD-IL-12 (i.t., n=10), 333 pmol IL-7-CBD (i.t., n=10), or 333 pmol IL-7-CBD (i.t.) + 33.3 pmol CBD-IL-12 (i.t., n=10) on day 7, 13, 19, and 25.

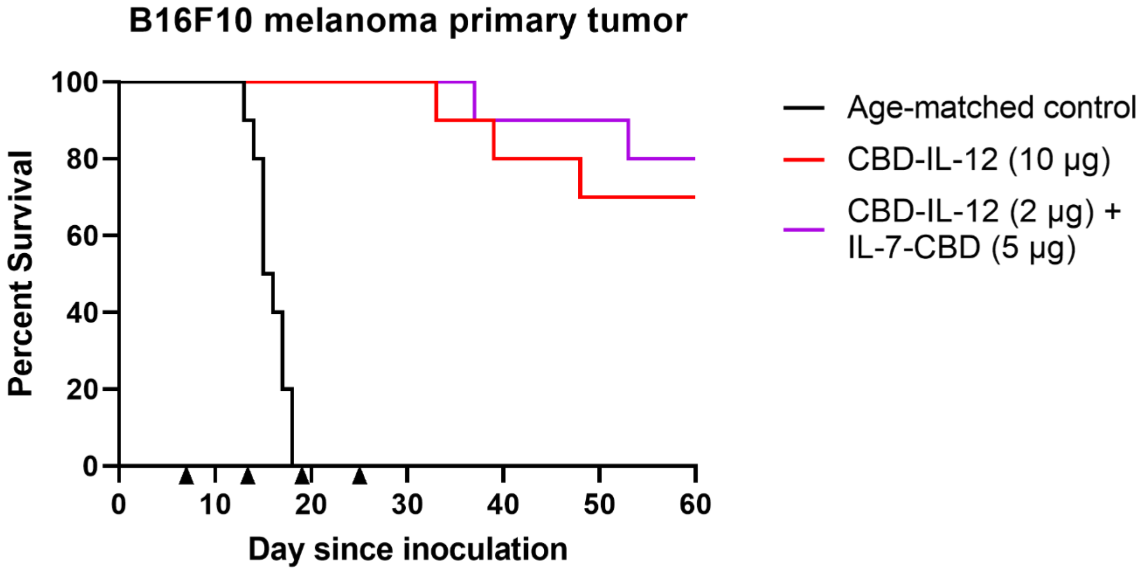

Supplementary Fig. 20. Percent survival of intratumorally administered high-dose CBD-IL-12 (10 µg) and low-dose CBD-IL-12 + IL-7-CBD in B16F10 model. C57BL/6 mice were inoculated with 5 x 10^5 B16F10 melanoma intradermally and injected with 30 µL PBS (i.t., n=10), 166.5 pmol CBD-IL-12 (i.t., n=10), or 33.3 pmol CBD-IL-12 (i.t.) + 333 pmol IL-7-CBD (i.t, n=10) on day 7, 13, 19, and 25.

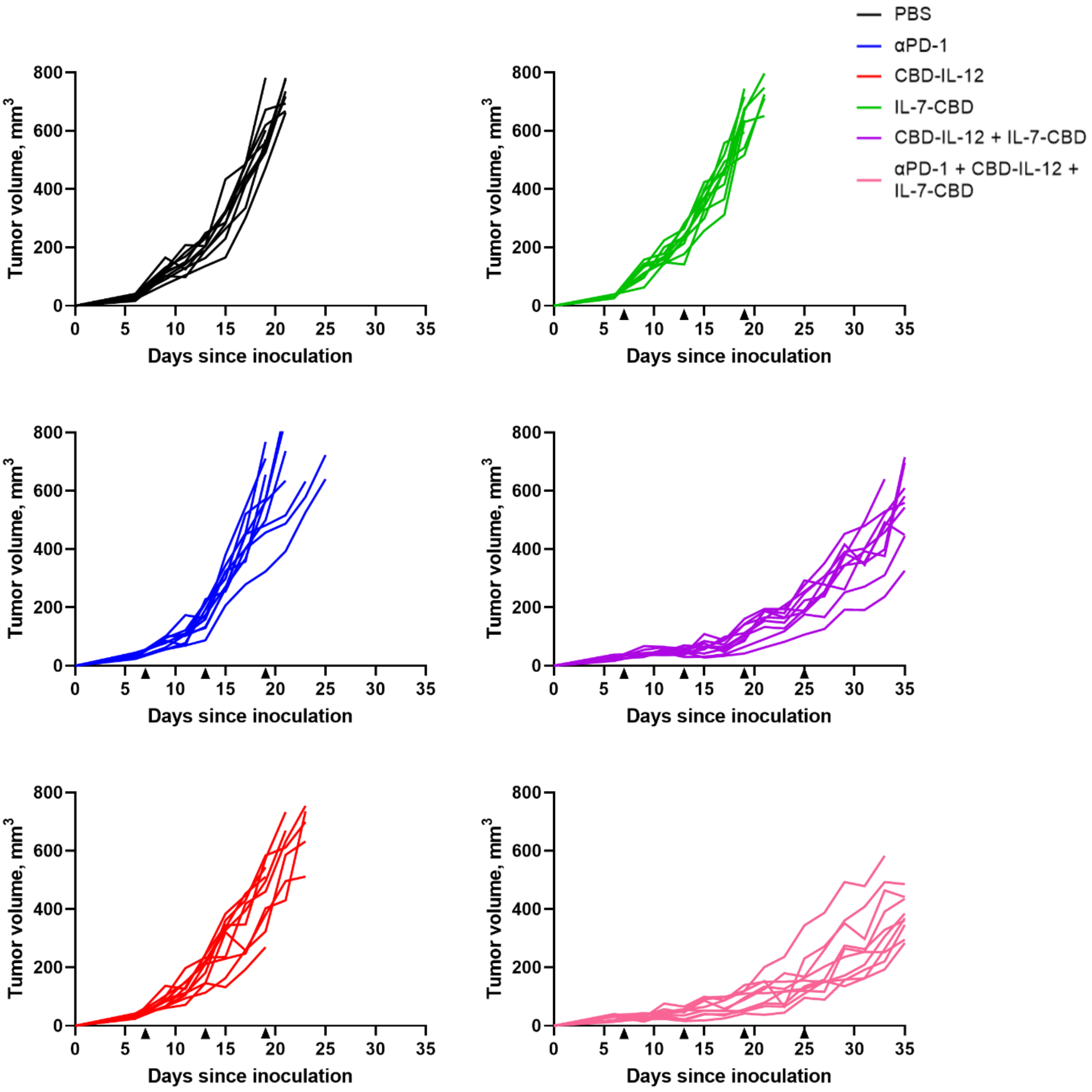

Supplementary Fig. 21. Combination therapy has a synergistic effect with αPD-1, and IL-7-CBD + CBD-IL-12 + αPD-1 significantly increases therapeutic scores in poorly immunogenic and CPI-unresponsive 4T1 breast cancer model. Balb/C mice were inoculated with 5 x 10^5 4T1 breast cancer cells into the mammary fat pad on day 0 and injected with 100 µg αPD-1 (i.p., n=10), 83.3 pmol CBD-IL-12 (i.t., n=10), 666 pmol IL-7-CBD (i.t., n=8), or 83.3 pmol CBD-IL-12 (i.t.) + 666 pmol IL-7-CBD (i.t., n=10) on days 7, 13, 19, and 25.

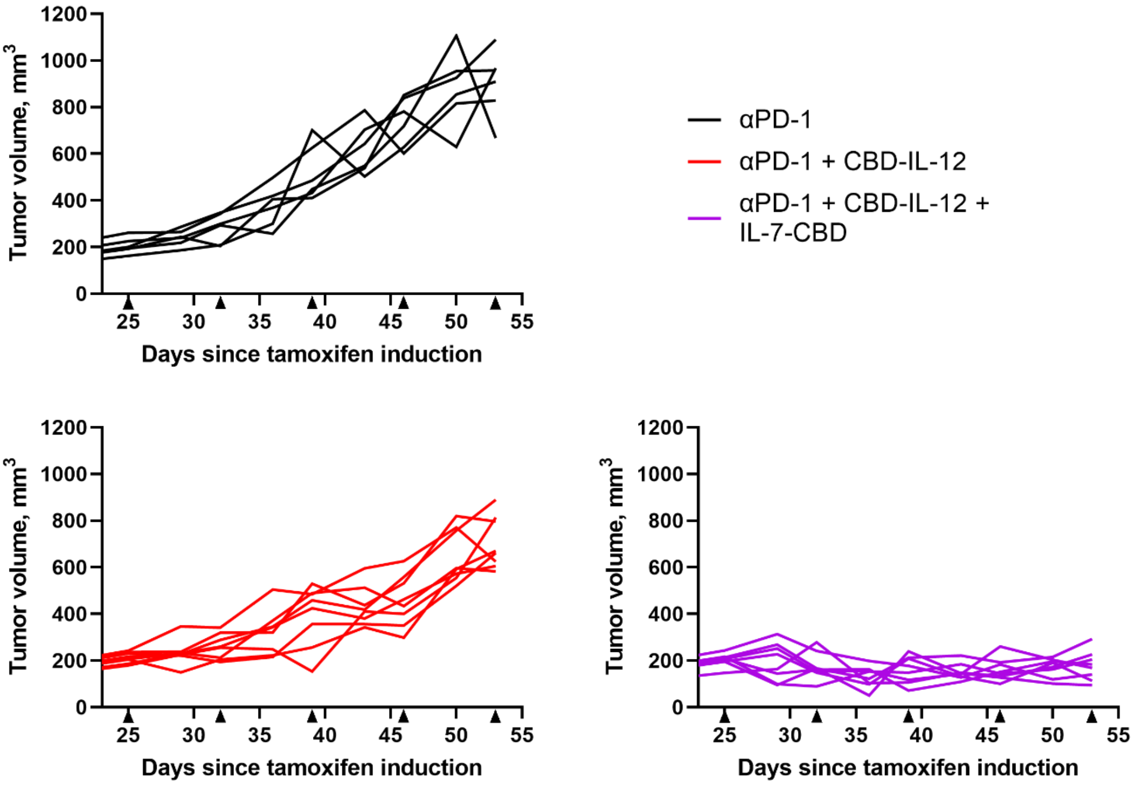

Supplementary Fig. 22. Combination therapy potently synergizes αPD-1, and IL-7-CBD + CBD-IL-12 + αPD-1 significantly increases therapeutic scores in poorly immunogenic and genetically engineered Braf^{V600E}/Pten$^{-/-}$/βCAT^{STA} melanoma model. Braf^{V600E}/Pten$^{-/-}$/βCAT^{STA} mice were applied 50 µg of 4-OH-tamoxifen on the back skin and treated with either 100 µg αPD-1 (i.p., n=6), 100 µg αPD-1 (i.p.) + 33.3 pmol CBD-IL-12 (i.v., n=8), or 100 µg αPD-1 (i.p.) + 33.3 pmol CBD-IL-12 + 666 pmol IL-7-CBD (i.v., n=8).

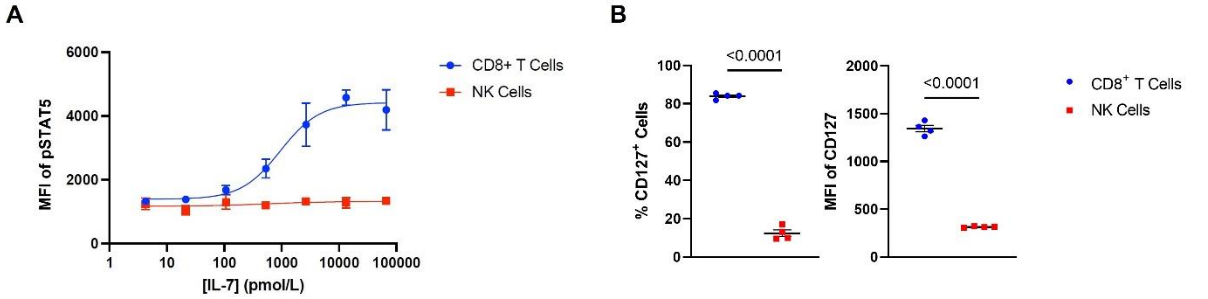

Supplementary Fig. 23. Dose-dependent phosphorylated STAT5 with mouse IL-7 and IL-7 receptor (CD127) expression level in mouse CD8$^+$ T cells or NK cells. Mouse CD8+ T cells or NK cells were isolated from the mouse spleen. **A.** The cells were treated with various concentrations of mouse IL-7 for evaluating STAT5 phosphorylation. **B.** The cell surface markers were stained for evaluating IL-7 receptor (CD127) expression level. Statistical analyses were performed using one-way ANOVA tests or t-tests. (*: *0.05>P*, **: *0.01>P*, and ***:*0.001>P*)

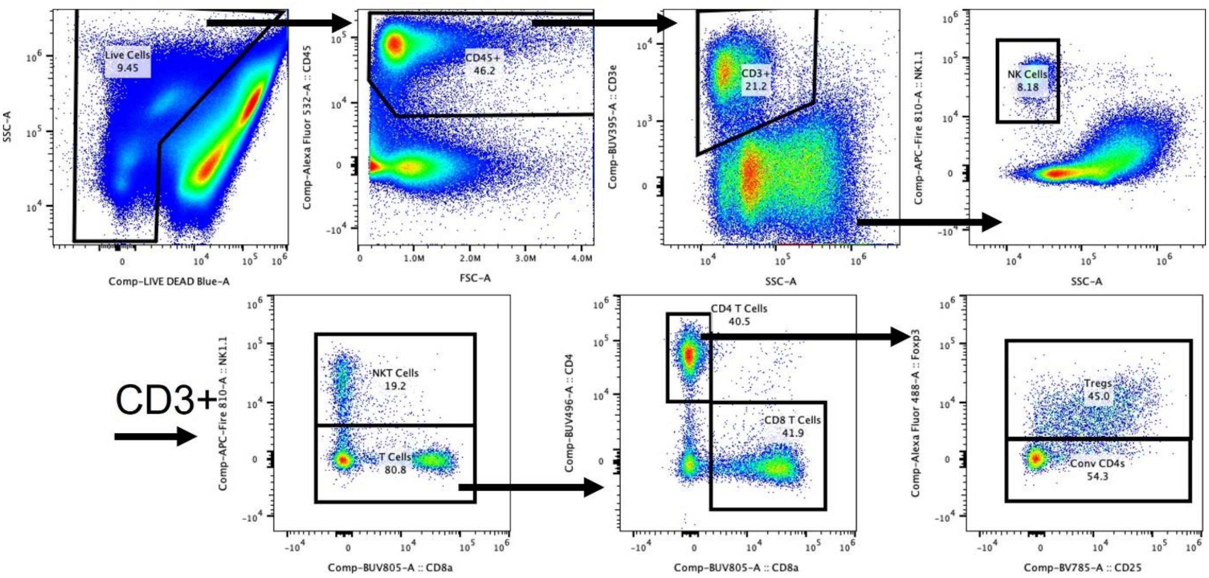

Supplementary Fig. 24. Representative gating strategy for gating CD8 T cells in the tumor.
